# Human Induced Pluripotent Stem Cell-Derived Macrophages Share Ontogeny with *MYB*-Independent Tissue-Resident Macrophages

**DOI:** 10.1016/j.stemcr.2016.12.020

**Published:** 2017-01-19

**Authors:** Julian Buchrieser, William James, Michael D. Moore

**Affiliations:** 1 Sir William Dunn School of Pathology, University of Oxford, South Parks Road, Oxford OX1 3RE, UK

**Keywords:** human iPSC macrophages, MYB, RUNX1, SPI1, hematopoiesis

## Abstract

Tissue-resident macrophages, such as microglia, Kupffer cells, and Langerhans cells, derive from *Myb*-independent yolk sac (YS) progenitors generated before the emergence of hematopoietic stem cells (HSCs). *Myb*-independent YS-derived resident macrophages self-renew locally, independently of circulating monocytes and HSCs. In contrast, adult blood monocytes, as well as infiltrating, gut, and dermal macrophages, derive from *Myb*-dependent HSCs. These findings are derived from the mouse, using gene knockouts and lineage tracing, but their applicability to human development has not been formally demonstrated. Here, we use human induced pluripotent stem cells (iPSCs) as a tool to model human hematopoietic development. By using a CRISPR-Cas9 knockout strategy, we show that human iPSC-derived monocytes/macrophages develop in an *MYB*-independent, *RUNX1-*, and *SPI1* (*PU.1*)-dependent fashion. This result makes human iPSC-derived macrophages developmentally related to and a good model for *MYB*-independent tissue-resident macrophages, such as alveolar and kidney macrophages, microglia, Kupffer cells, and Langerhans cells.

## Highlights

• Human iPSC-derived macrophages are *MYB* independent but *RUNX1* and *SPI1* dependent
• Transcription factor dependence genetically uncovers ontogeny of hiPSC macrophages

## Teaser

Macrophages are highly plastic multifunctional immune cells that play an essential role in mammalian tissues and their dysregulation is implicated in many pathological conditions. In this article, Buchrieser and colleagues define that iPSC-derived macrophages are of the same ontogeny as mouse tissue-resident macrophages, and are *MYB* independent. This work lays the foundation for the generation of authentic tissue-specialized macrophage subtypes.

## Introduction

Adult murine macrophages, in contrast to most other adult hematopoietic cells which renew from hematopoietic stem cells (HSCs) (Gekas et al., 2005), can derive from all three temporally and spatially distinct hematopoietic waves arising in the mouse embryo. A first wave occurs between E7.0 and E7.5 in the blood islands of the yolk sac (YS), producing *Myb*-independent nucleated erythrocytes, megakaryocytes, and macrophages (Mucenski et al., 1991). From E8.25, a second wave of hematopoietic cells emerge in the YS producing erythromyeloid progenitors (EMPs) (Palis et al., 1999, Palis et al., 2001), via a *Runx1*-dependent endothelial to hematopoietic transition (EHT) (Chen et al., 2009), that are capable of monocyte, macrophage, granulocyte, megakaryocyte, and erythrocyte differentiation. The presence of some kit+ EMPs (Schulz et al., 2012) and CD11 b^*high*^F4/80^*low*^ monocytes in the fetal liver of *Myb*^−/−^ mouse embryos (Gomez Perdiguero et al., 2015) suggests that EMP-derived monocytes and macrophages can develop independently of the transcription factor *Myb*. The third wave, from E10.5, consists of HSCs that are generated in the aorto-gonado-mesonephros (AGM) region of the embryo through *Runx1*-dependent EHT (Chen et al., 2009). HSCs depend on the transcription factor *Myb* for their maintenance and self-renewal (Lieu and Reddy, 2009, Schulz et al., 2012). While *Myb* is differentially required for different macrophage populations, primitive erythrocytes are the only cells that still arise in *Runx1*^−/−^ (Okada et al., 1998, Okuda et al., 1996, Wang et al., 1996) or *Spi1*^−/−^ (*Pu.1*) embryos (Scott et al., 1994). Murine YS-derived macrophages and fetal monocytes seed most tissues before HSC-derived definitive hematopoiesis (Ginhoux et al., 2010, Hoeffel et al., 2015, Kierdorf et al., 2013, Gomez Perdiguero et al., 2015, Schulz et al., 2012, Sheng et al., 2015) where, with the exception of dermal (Tamoutounour et al., 2013), gut (Bain et al., 2014), and a fraction of cardiac macrophages (Epelman et al., 2014), they self-renew throughout life with minor contribution from adult blood monocytes (Epelman et al., 2014, Hashimoto et al., 2013).

Our knowledge of human embryonic myeloid development and the ontogeny of tissue-resident macrophages is much more limited due to the difficulty obtaining human embryos at such an early developmental stage (Ivanovs et al., 2011). That said, several human studies are consistent with those in the mouse, in that most resident macrophage populations are independent of adult bone marrow (BM)-derived monocytes. Dermal macrophages remain of recipient origin for a prolonged time after HSC transplantation (Haniffa et al., 2009). After human hand allograft, Langerhans cells in the transplanted limb are still of donor origin 10 years post-transplantation (Kanitakis et al., 2011). Moreover, patients harboring mutations of *GATA-2* or *IRF8* have normal numbers of Langerhans cells and macrophages in the absence of circulating monocytes (Bigley et al., 2011, Bigley and Collin, 2011).

Human embryonic and induced pluripotent stem cells (hESCs/iPSCs) have been extensively studied with the aim of generating true transplantable HSCs with limited success (Ackermann et al., 2015), and although CD14^+^ macrophages can be easily derived from iPSCs (Karlsson et al., 2008, van Wilgenburg et al., 2013), their ontogeny is currently unknown. Klimchenko et al. (2011) have shown that hESC-derived monocytes/macrophages closely resemble, both transcriptionally and functionally, fetal-liver-derived monocytes/macrophages from first-trimester fetuses. Vanhee et al. (2015) recently showed, using an *MYB*-eGFP reporter hESC line, that CD14^+^ macrophages were generated through a CD34^+^CD43^+^GFP^−^ hematopoietic precursor. Although these observations are suggestive of an *MYB*-independent fetal-like ontogeny of human iPSC-derived monocytes/macrophages, no study has formally investigated the transcription factor requirement of human iPSC myelopoiesis.

Our laboratory has developed a feeder-free embryoid body (EB)-based protocol for the generation of a homogeneous monocyte/macrophage population from hESCs/iPSCs (Karlsson et al., 2008, van Wilgenburg et al., 2013). These cells express classical mononuclear phagocyte markers (CD14^*high*^CD16^*low*^CD163^+^CD11b^+^), are morphologically similar to blood-derived monocytes/macrophages, and display a macrophage cytokine expression profile while resting or activated using classical (interferon-γ/lipopolysaccharide [LPS]) or alternative activation (interleukin [IL]-4) (van Wilgenburg et al., 2013). In previous studies, macrophages produced using this protocol have been shown to display oxidative burst capacity (Flynn et al., 2015), high phagocytic capacity, and cytokine response on activation and are infectible by HIV-1 (van Wilgenburg et al., 2014), dengue virus (Cowley et al., 2012), influenza virus (van Wilgenburg et al., 2016), *Leishmania* (E. Gluenz, unpublished) and *Mycobacterium tuberculosis* (M. Gutierrez, unpublished). Although these monocytes/macrophages have been extensively studied, mapping their identity onto human hematopoietic development has been hindered by the limited data on human embryos, the lack of definitive human phenotypic markers discriminating tissue-resident macrophages from fetal monocytes or adult blood monocytes, as well as the lack of anatomical location in an iPSC system; thus we set out to genetically define their ontogeny.

With the aim of studying the requirement of *MYB*, *RUNX1*, and *SPI1* in the in vitro differentiation of these monocytes/macrophages from human iPSCs and mapping human hematopoiesis onto that of the mouse, we established knockout iPSC lines for each of these genes using the CRISPR-Cas9 system. We show that the monocytes/macrophages produced are *MYB* independent but *RUNX1* and *SPI1* dependent, which would tie them to *MYB*-independent myelopoiesis in the YS.

## Results

### iPSC-Derived Monocyte/Macrophage Development Is *MYB* Independent but *RUNX1* and *SPI1* Dependent

**Figure 1:**
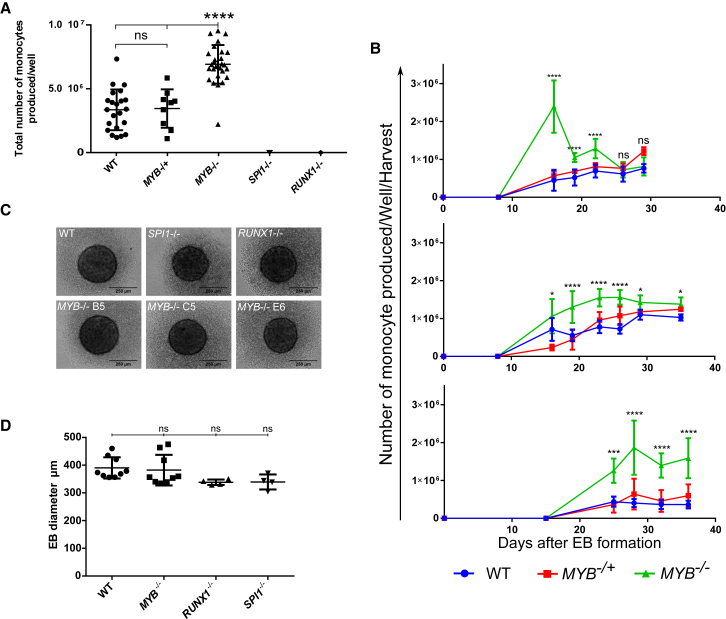
Monocyte/Macrophage Production Capacity of WT, *MYB*^*−/+*^, *MYB*^*−/−*^, *RUNX*^*−/−*^, and *SPI1*^*−/−*^ iPSCs (A) Total number of CD14^+^ cells produced per well containing eight EBs over a period of 30 days, plotted with mean and SD, three independent experiments, number of total wells: WT n = 22 (from three independent clones), *MYB*^*−/−*^ n = 27 (from three clones), *MYB*^*−/+*^ n = 9 (from one clone), *SPI1*^*−/−*^ n = 9 (from one clone), and *RUNX1*^*−/−*^ n = 9 (from one clone). Cell counts have been normalized to the CD14^+^ percentage of each replica (over 90% of the cells produced were CD14^+^ for each well independently of genetic modifications). Statistical comparisons were performed using a nonparametric Mann-Whitney test, ^∗∗∗∗^p < 0.0001. (B) Noncumulative production of monocytes per well over a period of 30 days of the three independent experiments shown in (A). Each time point represents the mean number of CD14^+^ cells harvested per well of *MYB*^*−/−*^ (n = 9), WT (n = 6), and *MYB*^*−/+*^ (n = 3) iPSCs. Error bars denote SD. Statistical comparisons were done using a two-way ANOVA, ns, nonsignificant, ^∗^p < 0.05, ^∗∗∗^p < 0.001, ^∗∗∗∗^p < 0.0001. (C) Representative image of WT, *MYB*^*−/−*^, *MYB*^*−/+*^, *RUNX1*^*−/−*^, and *SPI1*^*−/−*^ EBs after 1 day of differentiation. (D) Mean diameter with SD of WT, *MYB*^*−/−*^, *RUNX1*^*−/−*^, and *SPI1*^*−/−*^ EBs; each data point represents the mean diameter of one independent experiment (n = 16). Diameter was calculated using ImageJ, and statistical comparisons were performed using a nonparametric one-way ANOVA comparing the mean of each column with the mean of the WT control column.

We established knockout iPSC lines of *MYB*, *RUNX1*, and *SPI1* using a dual-guide RNA (gRNA)-targeting strategy (Figures S1–S4). To investigate the capacity of *MYB*^*−/−*^, *MYB*^*−/+*^, *SPI1*^*−/−*^, and *RUNX1*^*−/−*^ iPSCs to undergo myelopoiesis, we differentiated the iPSCs to monocytes/macrophages using our EB differentiation protocol (Karlsson et al., 2008, van Wilgenburg et al., 2013). Over a period of 30 days, wild-type (WT) and *MYB*^*−/+*^ iPSCs produced an average of 3 × 10^6^ monocytes/macrophages per well containing eight EBs, suggesting *MYB* haploinsufficiency had no effect on monocyte/macrophage commitment (Figure 1A). Interestingly, *MYB*^*−/−*^ iPSCs were capable of myeloid differentiation and produced 2-fold more CD14^+^ cells than WT and *MYB*^*−/+*^ (Figure 1A). When plotted as a noncumulative production of CD14^+^ monocytes/macrophages over time, it is apparent that *MYB*^*−/−*^ iPSCs produce significantly more monocytes/macrophages than the WT control or *MYB*^*−/+*^ iPSCs during the first weeks of production (Figure 1B). In contrast, *SPI1*^*−/−*^ and *RUNX1*^*−/−*^ iPSCs were unable to produce any CD14^+^ monocytes/macrophages (Figure 1A), although the EBs increased in size as expected and were comparable in their morphology when compared with WT or *MYB*^*−/−*^ EBs (Figures 1C and 1D).

### *MYB*^*−/−*^ iPSC-Derived Monocytes/Macrophages Display No Major Phenotypic or Functional Defects and Show a Similar Tissue-Resident Transcriptional Signature to WT Cells

**Figure 2:**
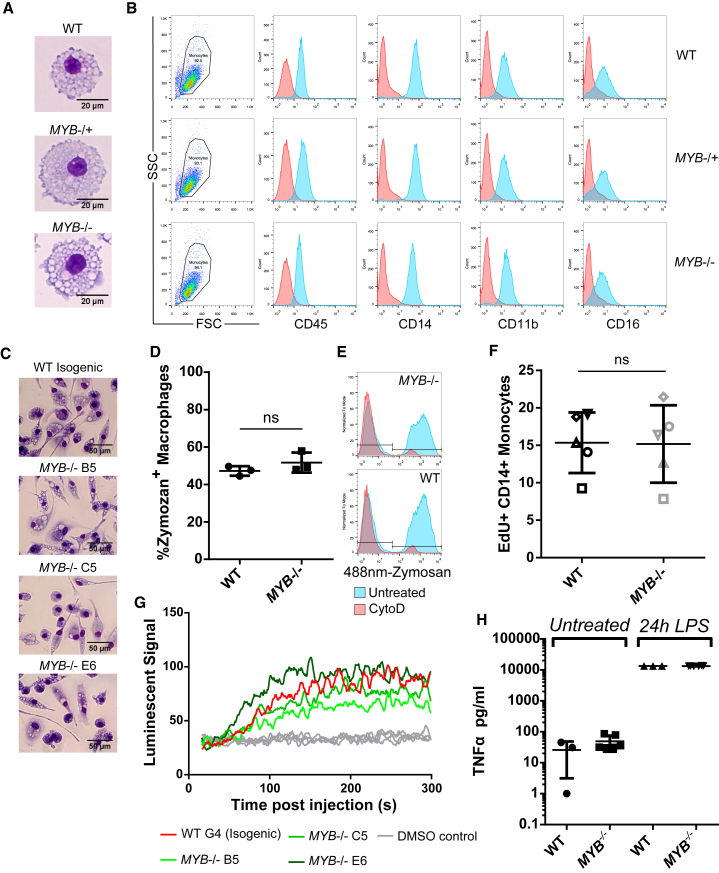
*MYB*^*−/−*^ Monocytes/Macrophages Display No Phenotypic or Functional Differences Compared with WT Control (A) Representative images of eosin and methylene blue stain of cytospined WT, *MYB*^*−/+*^, and *MYB*^*−/−*^ monocytes/macrophages on the day of harvest. (B) Flow cytometry staining of monocytes/macrophages for common myeloid cell surface makers, showing live cell gate on the left and histogram plots on the right, antibody staining (blue) and isotype (red). (C) Representative images of eosin and methylene blue stain of WT, *MYB*^*−/+*^, and *MYB*^*−/−*^ monocytes/macrophages differentiated for 7 days in M-CSF. (D) Phagocytosis assay measuring the fluorescently labeled zymosan uptake by 1 week differentiated WT and *MYB*^*−/−*^ macrophages. Cells were analyzed by flow cytometry and the percentage of AlexaFluor-488-zymosan^+^ macrophages was plotted. The experiment was run in triplicate, and statistical comparisons were done using an unpaired t test. Error bars denote SD between replicats. (E) Representative histograms of WT and *MYB*^−/−^ iPSC-derived monocyte/macrophages of AlexaFluor-488-zymosan uptake, untreated (blue) and cytochalasin D-treated negative control (red). (F) First harvest monocytes/macrophages pulsed with EdU and CD14^+^EdU^+^ cells that had undergone DNA replication were detected by flow cytometry after Click-iT staining. Five independent experiments are shown with mean and SD (symbols represent paired experiments), where *MYB*^*−/−*^ represents the pooled results obtained for three different *MYB*^*−/−*^ iPSC clones and WT the pooled result obtained from technical triplicates from a single clone. Statistical comparisons were done using a paired t test. (G) Monocytes/macrophages from WT and *MYB*^*−/−*^ iPSCs were treated with luminol reagent in the presence or absence of PMA stimulation. Individual wells were monitored for light released at 1 s intervals for 300 s in triplicate using a PHERAstar FS (BMG Labtech). The mean of three wells was normalized to the average of the 5 s before luminol addition and are plotted with smoothing for clarity (average of six neighboring data points). (H) ELISA measuring the TNF-α released by 1 week differentiated macrophages after 24 hr LPS stimulation compared with untreated controls. Results are plotted with mean and SD. No significant difference was observed using one-way ANOVA between WT and *MYB*^−/−^ cells.

**Figure 3:**
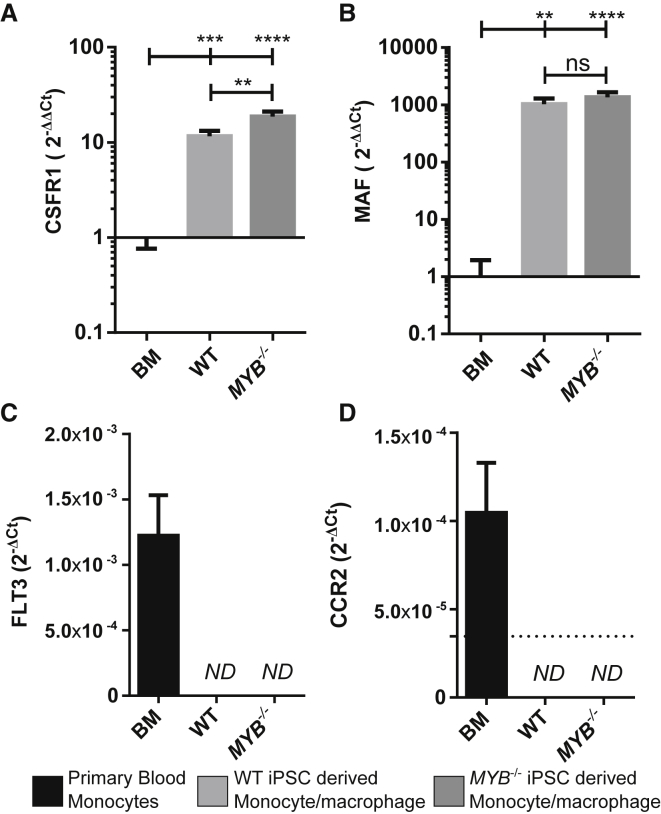
iPSC-Derived Monocytes/Macrophages Display a Similar mRNA Expression to Mouse *MYB*-Independent Macrophages Relative expression of (A) *CSFR1*, (B) *MAF*, (C) *FLT3*, (D) *CCR2* mRNA in primary blood monocytes (3 donors, n = 3), WT iPSC-derived monocytes/macrophages (three independent differentiations, n = 3), and *MYB*^−/−^ iPSC-derived monocytes/macrophages (two independent differentiations, three clones, n = 6). (A and B) Expression data were normalized to *EF1α* endogenous control, and relative mRNA expression to blood monocytes (2^−ΔΔCT^) is plotted. (C and D) C_T_ values of *CCR2* and *FLT3* were undetectable in all iPSC samples; therefore, the relative quantity of mRNA normalized to *EF1α* endogenous control is plotted (2^−ΔCT^). The dotted line represents the limit of detection equal to the 2^−ΔCT^ of the no-template control. Error bars denote SD. Statistical comparisons were done using a one-way ANOVA for each gene. ns, nonsignificant; ^∗∗^p < 0.01, ^∗∗∗^p < 0.001, ^∗∗∗∗^p < 0.0001. ND, non-detected/equal to no-template control.

As *MYB* is a major player in hematopoietic differentiation and hematopoietic cell function, we checked that the deletion of *MYB* did not affect the phenotype or function of the monocytes/macrophages generated. *MYB*^*−/−*^ and WT monocytes/macrophages showed no difference in morphology (eosin and methylene blue staining), phenotype (classical mononuclear phagocyte markers CD45, CD11b, CD14, and CD16), reactive oxygen species (ROS) production, tumor necrosis factor (TNF)-α release after LPS stimulation, and phagocytosis (zymosan uptake) (Figures 2A–2E, 2G, and 2H). While *MYB* knockout might affect macrophage function in a subtle way, *MYB*^−/−^ and WT monocytes/macrophages are overall highly similar. To ensure that in the absence of *MYB* the monocytes/macrophages produced are from the same developmental pathway as the WT cells, we analyzed the expression of *MAF*, *CSFR1*, *FLT3*, and *CCR2*, which are the most differentially expressed in mouse phenotypically YS-derived (CD11b^low^F4/80^high^) and blood-derived (F4/80^low^CD11b^high^) macrophages (Schulz et al., 2012). Relative expression of *MAF*, *CSFR1*, *FLT3*, and *CCR2* was quantified by RT-qPCR in primary blood monocytes and freshly harvested WT or *MYB*^−/−^ iPSC-derived monocytes/macrophages (Figures 3A–3D). *CSFR1* and *MAF* were expressed at significantly higher levels in iPSC-derived monocytes/macrophages than primary blood monocytes, while *FLT3* and *CCR2* were undetected in WT or *MYB*^−/−^ iPSC-derived monocytes/macrophages but were expressed in primary blood monocytes. These observations tie in with the mouse observation that *MAF* (involved in macrophage proliferation; Aziz et al., 2009) and *CSFR1* (macrophage colony stimulating factor [M-CSF] receptor) are highly expressed on YS-derived tissue-resident macrophages, while *FLT3* (expressed in pluripotent hematopoietic progenitors) and the chemokine receptor *CCR2* is low. A 2-fold increase of *CSFR1* was observed in *MYB*^−/−^ monocytes/macrophages, but this increase is comparatively small compared with the 10-fold difference with primary blood monocytes.

### *MYB* Knockout Results in Increased Number of Hematopoietic Progenitors within the EB

YS macrophages and fetal monocytes have a high-proliferative capacity compared with BM-derived monocytes (van de Laar et al., 2016), thus we set out to identify whether the increase in monocytes/macrophages from the *MYB*^−/−^ iPSC lines was due to the proliferation of the monocytes/macrophages during the first week of production (a time point at which we have observed the maximum rate of cell division; data not shown). To detect the rate of monocyte/macrophage division, we pulsed the monocytes with 5-ethynyl-2′-deoxyuridine (EdU) for 2 hr (Figure 2F). No significant difference was observed in monocytes/macrophages undergoing S phase (CD14^+^EdU^+^) between *MYB*^−/−^ and WT, suggesting that the increased monocyte/macrophage production is most likely due to an event upstream of the monocyte/macrophage differentiation stage.

**Figure 4:**
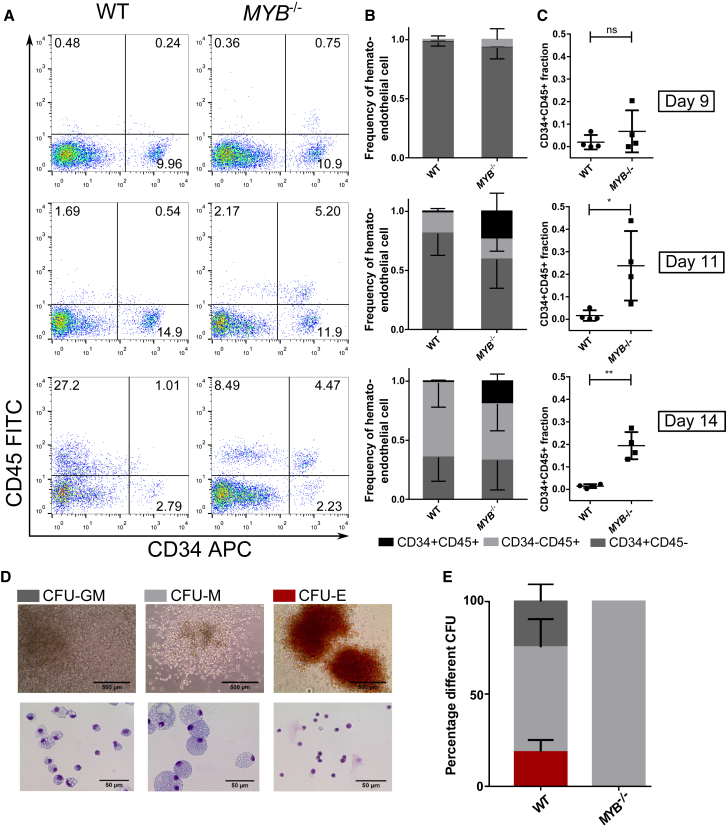
Study of the Progenitor Cells within the iPSC-Derived EBs (A) Representative plots of day 9, 11, and 14 EBs of WT and *MYB*^*−/−*^ iPSCs, which were enzymatically dissociated, stained for expression of CD34 and CD45, and analyzed by flow cytometry. Hemogenic and nonhemogenic-endothelial cells are CD34^+^CD45^−^, HPCs are CD34^+^CD45^+^, and differentiated hematopoietic cells are CD34^−^CD45^+^. (B) Relative proportion of CD34^+^CD45^+^, CD34^+^CD45^−^, and CD34^−^CD45^+^ populations on day 9, 11, and 14 normalized to the total number of single- and double-positive cells. Mean and SD of four repeats are plotted. (C) Relative proportion of CD34^+^CD45^+^ populations on day 9, 11, and 14. Statistical comparisons were done using a paired t test. ns, nonsignificant; ^∗^p < 0.05, ^∗∗^p < 0.01. (D) Image of the different colony types. The images on the left show a bright-field image of representative CFU-E, CFU-M, and CFU-GM WT colonies in methylcellulose media at day 14, while the images on the right show the cytospined and eosin and methylene blue-stained cells present within the three different types of colony. (E) Dissociated day 14 EBs were plated into H4434 MethoCult; after 14 days, colonies were scored. The percentage of each type of colony is displayed as mean with SD (WT n = 5, *MYB*^*−/−*^ n = 3 for each knockout clone). Presence of erythroid (CFU-E), granulocyte-macrophage (CFU-GM), and macrophage progenitors (CFU-M) can be detected in WT iPSC differentiation, whereas *MYB*^*−/−*^ iPSCs display only CFU-M potential. *RUNX1*^*−/−*^ and *SPI1*^*−/−*^ iPSCs did not generate any hematopoietic colonies. See also Figure S5.

To investigate whether *MYB*^−/−^ EBs generated more hematopoietic progenitors, we enzymatically dissociated EBs at various time points into single cells and stained for expression of the pan-hematopoietic marker CD45 and the endothelial/hematopoietic marker CD34. Hemogenic and non-hemogenic endothelial cells are labeled CD34^+^CD45^−^, hematopoietic progenitor cells are labeled CD34^+^CD45^+^, and committed hematopoietic cells are labeled CD34^−^CD45^+^ (Figure 4A). While the percentage of total CD34^+^ cells was similar between WT and *MYB*^−/−^ on days 9 and 11, very few CD34^+^CD45^+^ cells were detected in the WT condition, whereas a significant population of CD34^+^CD45^+^ cells (close to 20% of all single- and double-stained cells) was present in *MYB*^−/−^ (Figures 4B and 4C). This would suggest that *MYB*^−/−^ iPSCs undergo hematopoietic commitment earlier than WT iPSCs. Day 14 WT EBs show a major hematopoietic commitment, CD45^+^CD34^−^ representing 63% of the single- and double-stained population, while CD34^+^ CD45^+^ cells represent only 1.4% of the total (Figures 4B and 4C), indicating that the hematopoietic progenitor pool is small within the WT EBs. *MYB*^−/−^ EBs, on the other hand, display two distinct hematopoietic sub-populations at day 14; of the single- or double-stained cells, 19.5% were CD34^+^ CD45^+^ and 47% were CD45^+^ CD34^−^, indicating that a significantly larger hematopoietic progenitor cell pool is maintained within the *MYB*^−/−^ EBs. To further investigate the progenitors present at day 14 of differentiation, we stained EBs for CD41a and CD43, two markers present on lineage-restricted hematopoietic progenitors derived from an early EHT (Rafii et al., 2013) (Figure S5). CD41a^+^CD43^+^ hematopoietic progenitors can be detected in WT and *MYB*^−/−^ iPSC differentiation; they are absent from *RUNX1*^−/−^ and *SPI1*^−/−^ differentiations.

### iPSC-Derived Erythrocytes and Granulocytes Are Dependent on *MYB*, *RUNX1*, and *SPI1*

To investigate the progenitor potential of *MYB*^−/−^, *RUNX1*^−/−^, and *SPI1*^−/−^ iPSCs, EBs were differentiated for 14 days as for monocyte/macrophage differentiation, followed by enzymatic dissociation into single cells. The day 14 time point was chosen, as earlier time points (day 9 or day 11) derived only a small number of hematopoietic colonies. Single-cell suspensions were plated into MethoCult H4434, which supports the growth of erythroid progenitors (colony-forming unit [CFU]-E and burst forming unit [BFU]-E), granulocyte-macrophage progenitors (CFU-GM, CFU-G, and CFU-M), and multi-potential granulocyte, erythroid, macrophage, and megakaryocyte progenitors (CFU-GEMM). After 14 days of expansion, colonies were scored according to morphology. WT iPSCs generated CFU-E, CFU-GM, and CFU-M colonies, whereas *MYB*^−/−^ iPSCs generated only CFU-M colonies (Figures 4D and 4E). *RUNX1*^−/−^ and *SPI1*^−/−^ iPSC were unable to generate any hematopoietic colonies (data not shown).

## Discussion

**Figure 5:**
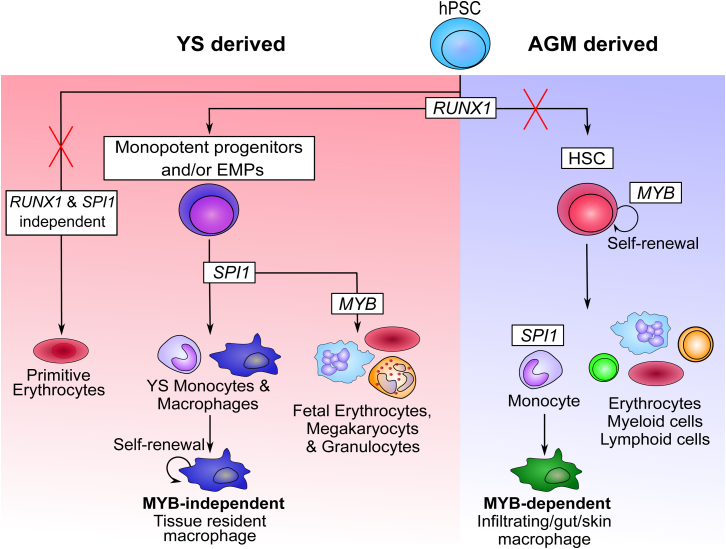
Schematic Representation of the Proposed Model of Lineage Commitment Occurring during Human iPSC Myelopoiesis In our protocol, when *RUNX1* is knocked out, no hematopoietic cells are observed, including primitive erythrocytes. This suggests the absence of unilineage primitive erythrocytes in this specific differentiation protocol (red cross indicates lack of production in our system) and confirms the mouse observation that *RUNX1* is required for all macrophage lineages. In contrast, *MYB* knockout only blocked erythroid and granulocyte colony-forming potential, which fits with the observation in the mouse that definitive erythrocytes and granulocytes require a functional *MYB* gene. *MYB* knockout does not block monocyte/macrophage differentiation, indicating that most iPSC-derived monocytes/macrophages generated in this study are *MYB* independent. The precursor of these monocytes/macrophages is still unknown, as macrophages could differentiate in a *MYB*-independent fashion from either unilineage primitive macrophage progenitors or EMPs. Furthermore, the fact that *MYB* is not required for myeloid differentiation in this EB differentiation protocol suggests the absence of definitive HSC commitment (red cross indicates lack of production in our system). Taken together, our results fit with the current observation in the mouse and suggest that *MYB*, *RUNX1*, and *SPI1* play a very similar role in mouse and human myeloid development.

In this report, we have used CRISPR-Cas9 to knock out key transcription factors in human iPSCs that are known to be involved in murine myeloid development. We used a feeder-free EB differentiation model to understand the developmental ontology and transcription factor requirement of in vitro generated monocytes/macrophages and thus map human iPSC myelopoiesis onto mouse hematopoietic development. Our results show that, using this protocol, iPSC-derived monocytes/macrophages are independent of the transcription factor *MYB*, which is required for definitive AGM hematopoiesis in the mouse (Mucenski et al., 1991, Mukouyama et al., 1999, Sumner et al., 2000), while being dependent on the transcription factor *RUNX1*, which is required for EHT (Chen et al., 2009), as well as *SPI1*, which is required for myeloid differentiation and plays a major role in many monocyte and macrophage functions (Anderson et al., 1998, Zakrzewska et al., 2010). The hematopoietic progenitors within the EBs are capable of macrophage, granulocyte, and erythrocyte potential, but in the absence of *MYB*, only macrophage colonies are detected by colony-forming assay in semi-solid media, suggesting that both CFU-E and CFU-GM are generated in an *MYB*-dependent fashion from EMPs. Furthermore, loss of erythrocyte colony potential in the *MYB* knockout iPSCs would indicate the absence of *RUNX1*- and *MYB*-independent primitive erythrocyte precursors within the EBs using this protocol. Taken together, our results suggest that iPSC-derived monocytes/macrophages can derive independently of *MYB* from *RUNX1*- and *SPI1*-dependent EMPs (Figure 5). The presence of primitive unilineage macrophage progenitors cannot be excluded without proper clonal analysis. Further investigation at the clonal level of the mesodermal, endothelial, and hematopoietic progenitors will be critical to define the relative contribution of monopotent primitive macrophage progenitors and EMPs to *MYB*-independent iPSC monocytes/macrophages.

While we cannot exclude the possibility that some *MYB*-dependent hematopoietic progenitor cells (HPCs)/HSCs are generated during normal WT iPSC differentiation, we would expect a reduction in monocyte/macrophage production if the majority of monocytes/macrophages were derived from *MYB*-dependent progenitors. In contrast, we observed an increase in production of monocytes/macrophages in the *MYB*^−/−^ iPSCs, without any major change in phenotype or function of the cells. Furthermore, using a similar EB-based human embryonic stem cell differentiation protocol, Vanhee et al. (2015) observed that multipotent HPCs expressing high levels of *MYB* were not generated in their cultures and that macrophages were generated from precursors not expressing detectable *MYB*. Combined with our data, this strongly suggests that in WT iPSC differentiation, most, if not all, monocytes/macrophages are produced in an *MYB*-independent fashion, and the contribution of *MYB*-dependent multilineage HPC/HSC-derived hematopoiesis in our EB-based monocyte differentiation protocol is negligible.

Interestingly, in addition to the increased monocyte/macrophage production, we observed an increased number of CD34^+^ CD45^+^ HPCs within *MYB*^−/−^ EBs. A very similar phenotype has been observed in mouse *Myb*^−/−^ ESC differentiation by Clarke et al. (2000). First, they observed that *Myb*^−/−^ ESCs were capable of macrophage and primitive erythrocyte colony formation, but the kinetics of formation was different between the control line and the *Myb*^−/−^ ESCs. The number of CFU-E was lower in *Myb*^−/−^ while the generation of CFU-M was increased at day 7 when compared with the WT control. Second, no BFU-E were generated by *Myb*^−/−^ ESCs, indicating a block to definitive erythrocyte production. Third, they observed a higher number of CD34^+^Sca-1^+^ HPCs within the *Myb*^−/−^ EBs, but these progenitors were unable to progress further in differentiation. This could be due to an increased hematopoietic commitment, progenitor proliferation, or an accumulation of committed erythrocyte/granulocyte progenitors that cannot progress further through differentiation due to the lack of *Myb*. It will be interesting to understand the precise mechanism of action underlying this increase in precursor cells and monocytes/macrophages.

With the mounting data suggesting that tissue-resident macrophages and BM monocyte-derived macrophages can play different roles in diseases such as cancer (Lahmar et al., 2015) and parasite infection (Rückerl and Allen, 2014), having access to authentic embryonic-derived monocytes and macrophages in vitro will be of considerable scientific value. Patient-derived tissue-resident macrophages are very difficult to obtain, are inherently genetically variable, and are notoriously difficult to genetically modify, making their study laborious and unreliable. On the other hand, iPSCs can be generated from a patient with a specific genetic background and can be modified by multiple mechanisms, such as lentiviral transduction or CRISPR-Cas9 gene editing. The demonstration that *MYB*-independent monocytes/macrophages can be generated in our differentiation protocol lays the foundation for their use in the development of reliable protocols for generating the tissue-specific subtypes of macrophages for the in vitro study of their role in pathology and homeostasis. Moreover, iPSC differentiation is a potential source of tissue-resident macrophages for cell therapy, as has recently been shown in the mouse with the use of murine pluripotent stem cell-derived *Myb*^−/−^ alveolar-like macrophages as a cell source for treating a mouse model of adenosine deaminase deficiency *(ADA*^−/−^) (Litvack et al., 2016).

## Experimental Procedures

### Human iPSC Culture

The human iPSC line AH016-03 was the target line for all gene editing in this study. The derivation and characterization of iPSC line AH016-03 has been previously published (Sandor et al., 2017), and was derived from a normal healthy donor, having given signed informed consent, which included derivation of human iPSC lines from skin biopsies (Ethics Committee: National Health Service, Health Research Authority, NRES Committee South Central, Berkshire, UK (REC 10/H0505/71)). The SNP datasets and the Illumina HT12v4 transcriptome array results of the parental cell line have been deposited in Gene Expression Omnibus under accession number GEO: GSM2055806. The SNP datasets of the genetically modified lines have been deposited in GEO: GSE93285. iPSCs were cultured in feeder-free conditions in mTeSR1 (STEMCELL Technologies) on Matrigel (Scientific Laboratory Supplies 354277). Cells were passaged with TrypLE Express (Gibco by Life Technologies) and plated in media containing 10 μmol/L Rho-kinase inhibitor Y-27632 (Abcam). The number of passages was kept to a minimum to reduce the likelihood of genetic change, and cells were frozen in SNP quality-controlled batches from which cells would be thawed for each experiment, to ensure consistency.

### EB Formation

Spin-EBs were formed using a 96-well ultra-low adherence plate (Costar 7007). iPSCs were washed with PBS and harvested by incubating the cells for 5 min at 37°C with 1 mL of warm TrypLE Express (Gibco by Life Technologies). Cells were counted, washed with PBS, and resuspended at a final concentration of 1.25 × 10^5^ cells/mL in EB media: mTeSR1 (STEMCELL Technologies), 50 ng/mL BMP-4 (GIBCO- PHC9534), 20 ng/mL stem cell factor (Miltenyi Biotec), 50 ng/mL vascular endothelial growth factor (GIBCO- PHC9394); 100 μL of cell suspension in EB media supplemented with 10 μmol/L Y-27632 was added per well, and the 96-well plate was centrifuged at 100 × *g* for 3 min and incubated for 4 days. EBs were fed at days 1 and 2 by aspirating 50 μL of medium and adding 50 μL of fresh EB medium.

### Myeloid Differentiation

After 4 days of EB differentiation, EBs were transferred into a six-well tissue-culture plate (8 EBs/well) (CorningCostar) and resuspended in monocyte/macrophage differentiation media consisting of X-VIVO-15 (Lonza), supplemented with 100 ng/mL M-CSF (Invitrogen), 25 ng/mL IL-3 (R&D), 2 mM glutamax (Invitrogen), 100 U/mL penicillin and 100 mg/μL streptomycin (Invitrogen), and 0.055 mM β-mercaptoethanol (Invitrogen). Two-thirds of the media was changed every 5 days. After the first production of iPSC-derived monocytes/macrophages, non-adherent monocytes/macrophages were harvested from the supernatant every week for staining and counting.

### Cell Count and Viability

Cells were counted using NC-3000 Viability and Cell Count Assays (Chemometec) according to the manufacturer's instructions.

### EdU Staining

Monocytes/macrophages were harvested and counted, and 1 × 10^5^ cells were transferred into a well of a 12-well plate without media change. Cells were pulsed with 10 μM EdU for 2 hr after which they were harvested and stained according to the manufacturer’s protocol for the Click-iT Plus EdU Flow Cytometry Assay Kits (Thermo Fisher).

### Eosin and Methylene Blue Staining

Freshly harvested monocyte (5 × 10^4^) cells were either centrifuged at 400 × *g* for 4 min onto a glass slide using a Cytospin 3 (Shandon) or further differentiated for 7 days on glass coverslips in macrophage differentiation media consisting of monocyte/macrophage differentiation media without IL-3 (X-VIVO-15 [Lonza], supplemented with 100 ng/mL M-CSF [Invitrogen], 2 mM glutamax [Invitrogen], 100 U/mL penicillin and 100 mg/μL streptomycin [Invitrogen], and 0.055 mM β-mercaptoethanol [Invitrogen]). Slides were air dried briefly and stained using an eosin and methylene blue staining kit (HemaGurr) as specified by the manufacturer’s protocol. Images were acquired using an EVOS inverted microscope.

### EB Dissociation and Colony-Forming Assay

Twenty-four EBs per condition were harvested using a 100-μm cell strainer, washed in PBS, and EBs were resuspended in 500 μL of Accumax Solution (Sigma). EBs were incubated for 5 min at 37°C, mechanical dissociation was performed by carefully pipetting up and down using a 200 μL pipette for 1 min, after which the EBs were incubated an extra 5 min at 37°C followed by mechanical dissociation for an additional minute. The resulting cell suspension was filtered using a 70 μm cell strainer before being centrifuged for 5 min at 400 × *g*. Colony-forming cell assay was performed according to the manufacturer's protocol using a cell concentration of 3 × 10^5^ cells/mL resulting in a final plating concentration of 3 × 10^4^ cells per 35 mm dish of MethoCult H4434 (STEMCELL Technologies). Colonies were scored by morphology 14 days after plating.

### Flow Cytometry Staining and Antibodies

Harvested monocytes/macrophages or single-cell suspension obtained from EB dissociation were pelleted at 400 × *g* for 5 min and washed once with PBS before being resuspended in 100 μL of fluorescence-activated cell sorting (FACS) buffer (PBS +10 μg/mL human serum IgG + 1% fetal bovine serum [FBS]). Cells were stained in FACS buffer + antibody (dilution 1:100) for 45 min at 4°C. Cells were then washed using PBS and resuspended in 2% formaldehyde before being analyzed using a FACSCalibur flow cytometer (BD Biosciences). The following antibodies have been used in this study: α-CD14-APC antibody (MEM-15; Immunotools; 21279146), IgG1-APC isotype (PPV-06; Immunotools; 21275516X2), α-CD34-APC (4H11; eBioscience; 17-0349-42), mouse IgG1κ-APC isotype (P3.6.2.8.1; eBioscience; 17-4714-41), α-CD45-fluorescein isothiocyanate (FITC) (MEM-28; ImmunoTools; 21270453X2), mouse IgG1-FITC isotype (PPV-06; ImmunoTools; 21275513X2), α-CD16-APC (LNK16; Immunotools; 21279166X2), α-CD11b-APC (ICRF44; BioLegend; 301309), and mouse IgG1-APC isotype (MOPC-21; BioLegend; 400119).

### Phagocytosis Assay

Harvested monocytes/macrophages were plated on tissue-culture plates in macrophage differentiation media for 7 days. On the day of the analysis, negative control wells were pretreated with 10 μM cytochalasin D for 1 hr. After pretreatment, all wells were fed with media containing two particles per cell of 488-zymosan (Z-23373, Thermo Fisher) and incubated for 30 min at 37°C. Macrophages were detached using 5 mM EDTA and 12 mM lidocaine (Sigma) in PBS and membrane-bound zymosan was quenched using 250 μg/mL trypan blue (Gibco) for 5 min before fixation in 2% formaldehyde. Stained cells were analyzed on a FACSCalibur flow cytometer (BD Biosciences).

### TNF-α ELISA

Harvested monocytes/macrophages were plated on tissue-culture plates in macrophage differentiation media for 7 days. Positive control wells were activated with LPS at 100 ng/mL for 24 hr. Cleared cell culture supernatants were diluted 1:5 and probed using the TNF-α ELISA Ready set go (88-7346-86, eBioscience) according to the manufacturer’s instructions.

### ROS Assay

Harvested monocytes were plated onto tissue-culture plates for 24 hr in macrophage differentiation media for 7 days, before analysis for ROS production. The luminol assay was used to identify ROS activity from macrophages, as previously described (Jiang et al., 2012), after stimulation with 200 ng/mL phorbol 12-myristate 13-acetate (PMA). Individual wells were monitored for light released at 1-s intervals for 300 s using a PHERAstar FS (BMG Labtech).

### RNA Extraction, Reverse Transcription, and qPCR

Peripheral blood of three healthy adult volunteers was collected according to University of Oxford OHS policy document 1/03, with signed informed consent. Peripheral blood mononuclear cells were isolated from blood by density gradient centrifugation with Ficoll-Paque PLUS (17-1440-03, GE Healthcare) and monocytes were sorted using CD14 MACS beads (130-050-201, Miltenyi). A total of 2 × 10^6^ iPSCs, freshly harvested iPSC-derived monocytes/macrophages, or primary blood monocytes were lysed using RLT buffer (QIAGEN) supplemented with 10 μL of β-mercaptoethanol. RNA extraction was performed using the RNeasy kit (QIAGEN) according to the manufacturer's protocol. Potential DNA contamination was removed by adding a step of Ambion TURBO DNA-free according to the manufacturer's protocol (Life Technologies). Reverse transcription was performed using the RetroScript (Ambion) kit or the high-capacity RNA-to-cDNA kit (Applied Biosystems) according to the manufacturer's protocol. qPCR was performed using Brilliant III SYBR (Agilent) on the Applied Biosystems StepOnePlus Real-Time PCR System. The following primers were used: EF1α forward (5′-CTG AAC CAT CCA GGC CAA AT-3′), EF1α reverse (5′-GCC GTG TGG CAA TCC AAT-3′), MYB forward (5′-GCC AAT TAT CTC CCG AAT CGA-3′), MYB reverse (5′-ACC AAC GTT TCG GAC CGT A-3′), CSFR1 forward (5′-TCC AAC ATG CCG GCA ACT A-3′), CSFR1 reverse (5′-GCT CAA GTT CAA GTA GGC ACT CTC T-3′), CCR2 forward (5′-GAC AGA GAC TCT TGG GAT GAC TCA-3′), CCR2 reverse (5′-ATC CTA CAG CCA AGA GCT ATG TGA-3′), FLT3 forward (5′-CAA ATC AGA TGT ACG TGG AC-3′), FLT3 reverse (5′-GCT GTA TCC GTT ATC AAG AC-3′), MAF forward (5′-GTA CAA GGA GAA ATA CGA GAA G-3′), and MAF reverse (5′-TAT GAA AAA CTC GGG AGA GG-3′). Actin-β control forward and reverse primers were purchased from Eurogentech.

### Immunostaining

Day 14 differentiated EBs were fixed in 4% paraformaldehyde, permeabilized in PBS + 0.3% Triton X-100 (Sigma), and blocked for 1 hr in blocking buffer (PBS + 10 μg/mL human serum IgG + 5% FBS). Samples were incubated overnight at 4°C in blocking buffer containing primary antibodies directly conjugated to FITC or APC. Antibodies used were α-CD43-FITC (1G10; BD Biosciences; 560978) and α-CD41a-APC (HIP8; BD Biosciences; 561852). Imaging was performed using an EVOS FL Auto Cell Imaging System using a ×10 or ×20 objective.

## Author Contributions

J.B. designed, performed, and analyzed experiments and wrote the manuscript; W.J. and M.D.M. designed and supervised the studies and edited the manuscript.

## Supplemental Information

Document S1. Supplemental Experimental Procedures and Figures S1–S5

Document S2. Article plus Supplemental Information
