# Supplementary material for: Human Induced Pluripotent Stem Cell-Derived Macrophages Share Ontogeny with MYB-Independent Tissue-Resident Macrophages
Source: Stem Cell Reports. 2017 Jan 19;8(2):334–45. doi: 10.1016/j.stemcr.2016.12.020 (PMC5312255; doi:10.1016/j.stemcr.2016.12.020)
Supplement: Document S1. Supplemental Experimental Procedures and Figures S1–S5 [file mmc1.pdf]

Stem Cell Reports, Volume *8*

Supplemental Information

Human Induced Pluripotent Stem Cell-Derived Macrophages Share Ontogeny with *MYB*-Independent Tissue-Resident Macrophages

Julian Buchrieser, William James, and Michael D. Moore

Supplementary Figures and legends

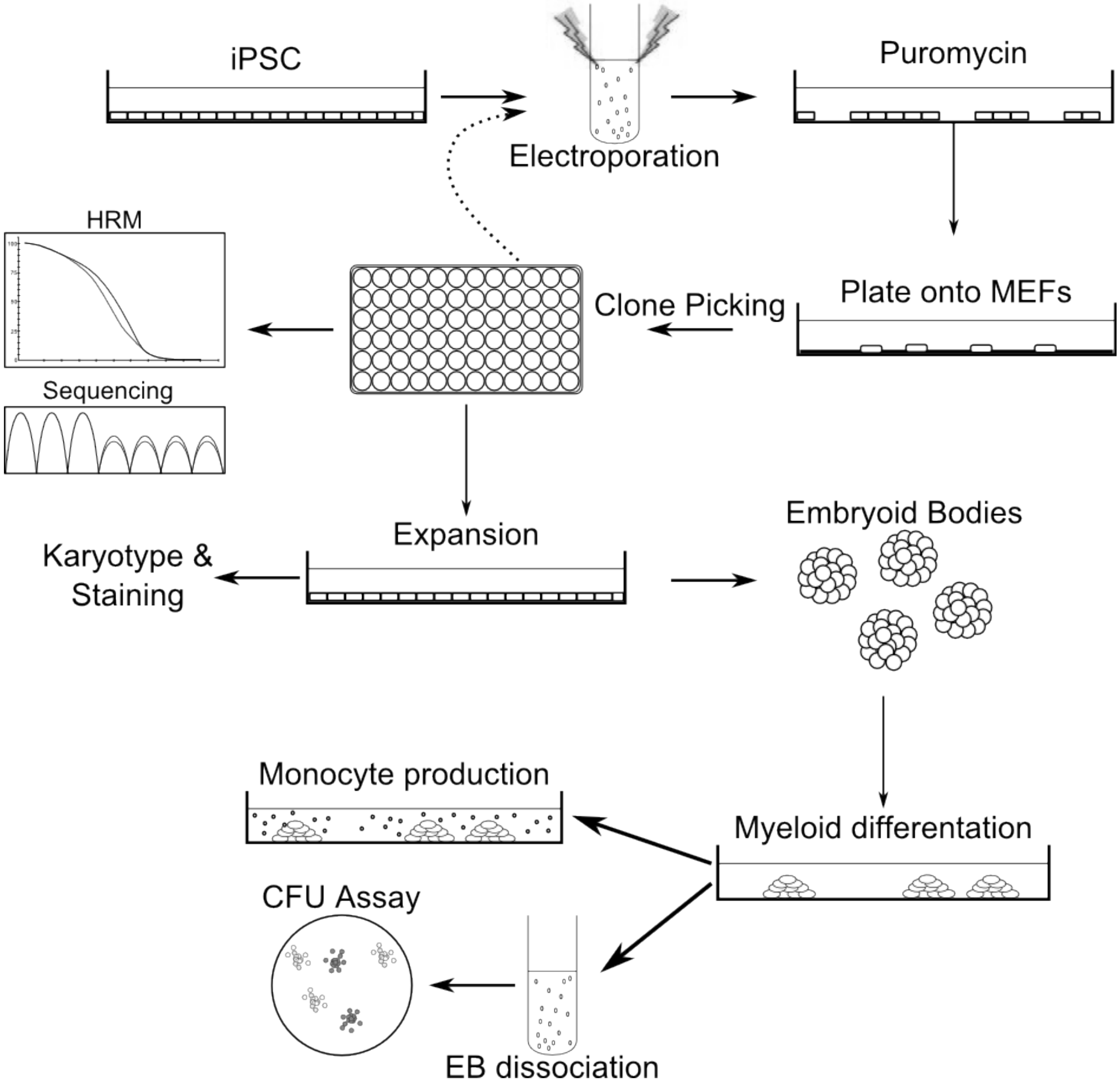

Supplementary figure S1. Schematic representation of the CRISPR-Cas9 based knock-out strategy in iPSCs. *Related to supplementary methods "Gene editing and single-cell cloning".* IPSCs generated from a healthy donor were cultured under feeder-free conditions, electroporated with two, pX462 plasmids for Cas9-gRNA-puromycin expression. Transfected cells were selected by transient puromycin treatment after which they were plated at low density onto mouse embryonic fibroblast feeder cells. After 7 days of expansion, individual single cell colonies were picked into a 96 well plate directly in feeder-free conditions and clones were expanded. IPSC clones were pre-screened for insertions or deletions in the targeted region using high resolution melt analysis (HRM)

(Supplementary Figure S2A) and sequenced to determine the exact sequence of each clone (Supplementary Figure S3C). Clones harbouring a single out of frame or a double out of frame deletion in the gene of interest were expanded, stained for pluripotency markers and karyotyped (Supplementary Figure S4). IPSC lines were then assessed for myeloid differentiation potential and hematopoietic colony formation capacity.

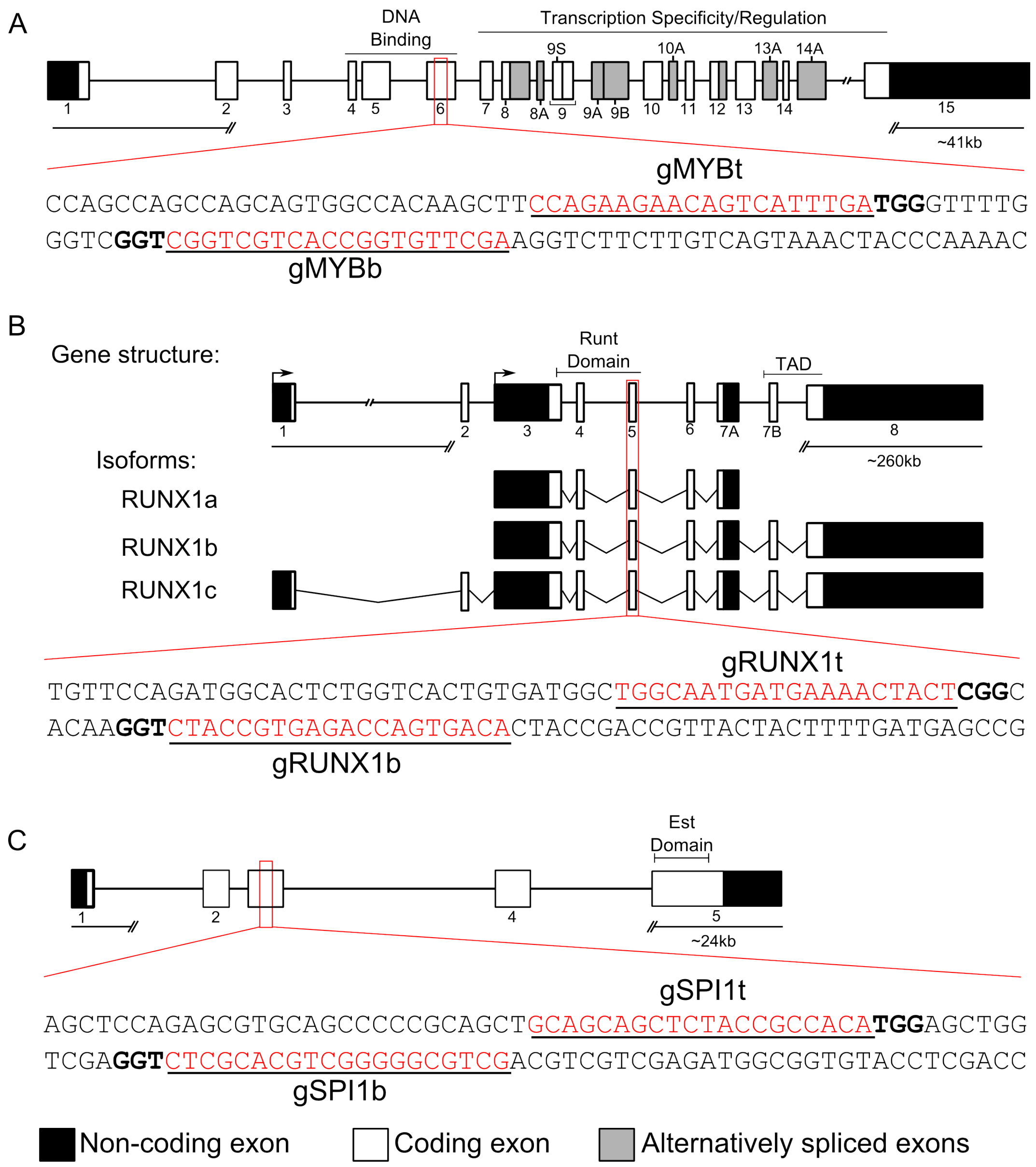

Supplementary figure S2. Exon structure, splice variants, major functional domains and CRISPR-Cas9 target site of the *MYB* (A), *RUNX1* (B) and *SPI1* (C) genes. *Related to supplementary methods "Gene editing and single-cell cloning".* For each gene an enhanced view of the target sequence is shown, CRISPR-Cas9 targeting site is shown in red and PAM sites in bold. *RUNX1* encodes for three major isoforms, RUNX1a, RUNX1b and RUNX1c (Osato, 2014).

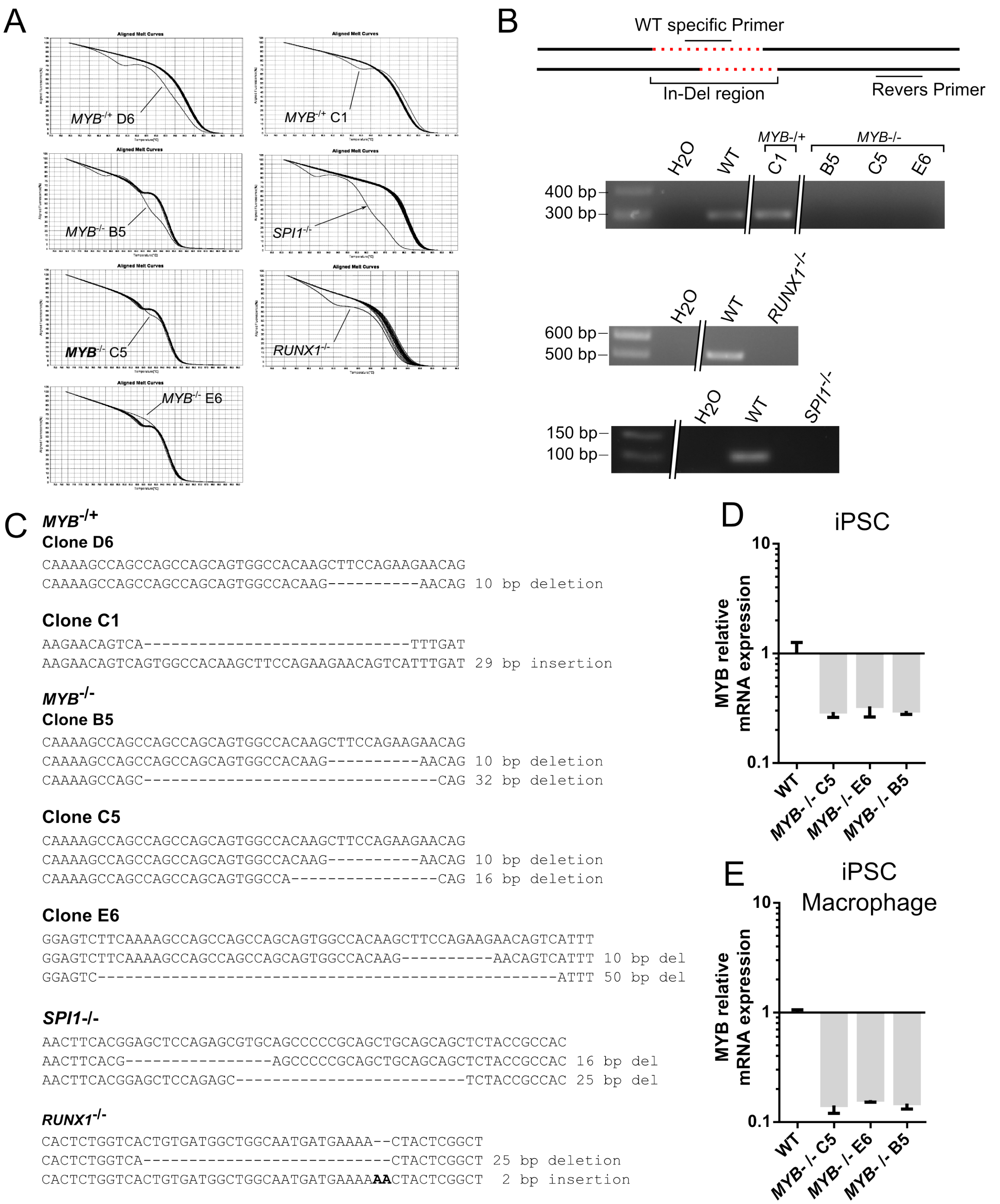

Supplementary figure S3. Genomic analysis of the knock-out iPSC clones. *Related to supplementary methods "Gene editing and single-cell cloning".* (**A**) High resolution melt curves obtained from the 7 different iPSC clones used in this study shown in comparison to the melt curves obtained from their respective unmodified parent lines. (**B**) Primers designed to specifically amplify wild type sequence and not knock-out sequence showed

total absence of amplification in all knock-out clones. First, a schematic representation of WT specific primer design, forward primers were designed to span the deletion and/or insertion present in the knock-out clones hindering binding of the primers and amplification of knock-out sequence but allowing the amplifying WT sequence. Second, *MYB* clones were tested using forward primer JB-107 (5'-TGGCCACAAGCTTCCAGAAG-3') and reverse primer JB-64 (5'-ACCATACCTACACCCTATCTACTTCAAAG-3'). *MYB* and WT clones amplified while all three *MYB* clones did not amplify any product. Third, *RUNX1* clone was tested using forward primer JB-110 (5'-GGCTGGCAATGATGAAACCT-3') and reverse primer JB-78 (5'-GATAGCCCACAGATCATACGTCAA-3'), the forward primer was designed with an extra mismatch to increase specificity as one allele of the *RUNX1* clone is a short 2 bp insertion. WT positive control DNA amplified while *RUNX1* didn't. Last, *SPI1* clone was tested, forward primer JB-108 (5'-GAGCTCCAGAGCGTGCAGCC-3') and reverse primer JB-92 (5'-CAGGAGGGCCCACAACAA-3'). WT positive control DNA amplified while *SPI1* didn't. (**C**) Sequence analysis of the different knock-out single iPSC clones showing both alleles compared to the original wild type sequence. Each complete knock-out clone harbours an out-of-frame deletion or insertion on both alleles, while single allele knock-out clone still harbour one wild type allele. (**D-E**) Relative expression of *MYB* mRNA in WT and *MYB*$^{-/-}$ iPSCs and iPSC-derived monocytes/macrophages showing an efficient knock-down of the mRNA levels of *MYB*.

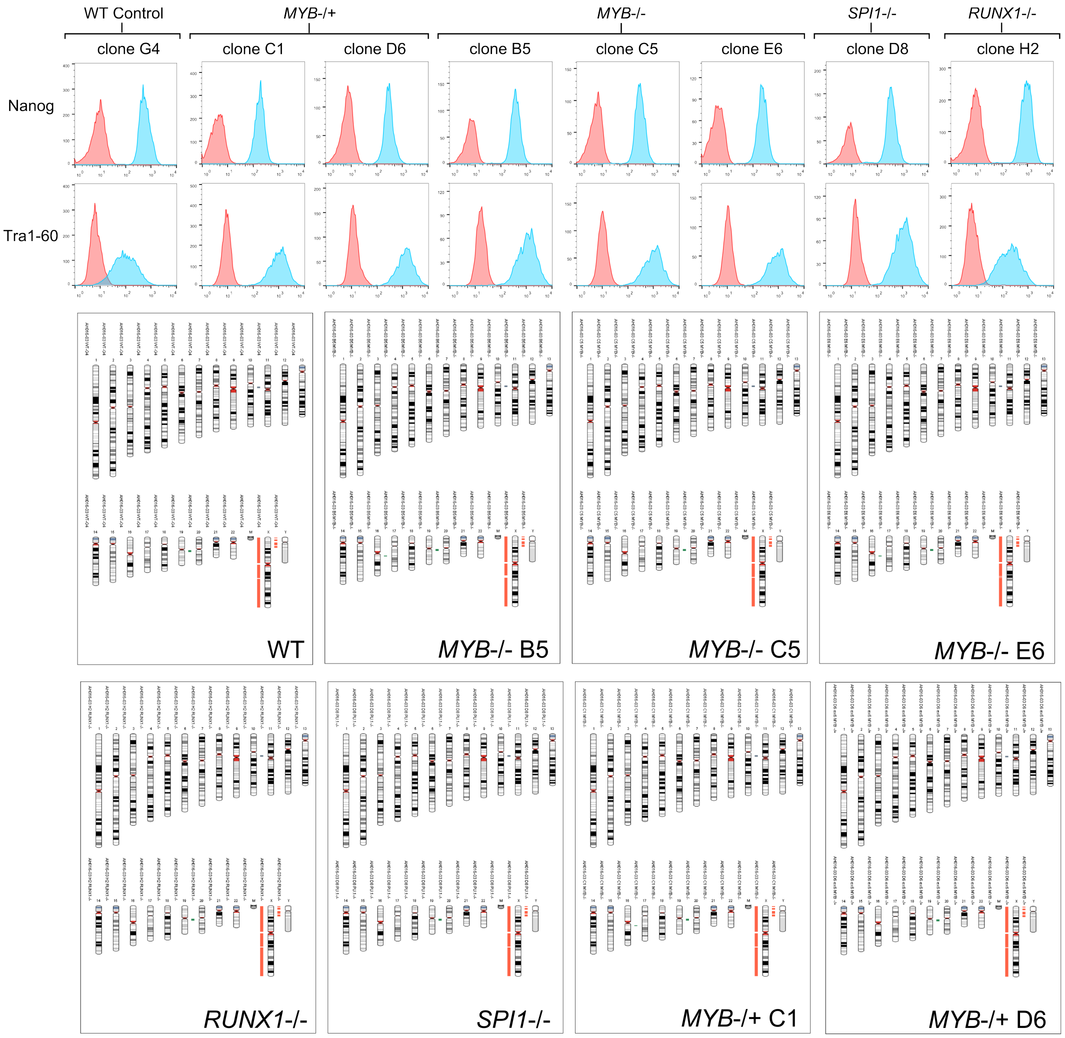

Supplementary figure S4. Gene-knock-out iPSC lines maintained pluripotency and karyotype. *Related to supplementary methods "Gene editing and single-cell cloning".* (**A**) iPSCs lines were stained for TRA-1-60 (1.5 mg/mL; α-TRA-1-60-AlexaFluor®488; Biolegend; 330614) and NANOG (0.3 mg/mL; α-NANOG-AlexaFluor®647; Cell Signaling Technologies; D73G4), flow cytometry analysis are shown as histograms, antibody staining (blue) and isotype (red). (**B**) DNA extracted from the iPSCs was karyotyped using a SNP array (Illumina OmniExpress24 chip covering w700,000 markers) and analysed using KaryoStudio (Illumina) to detect copy number variations across the genome. Red indicates a single copy of the SNPs (demonstrated by the single X copy in this male patient's DNA); gray indicates loss of heterozygosity; and green indicates duplications of a stretch of DNA (one amplification on ch20 was present on all iPSC clones).

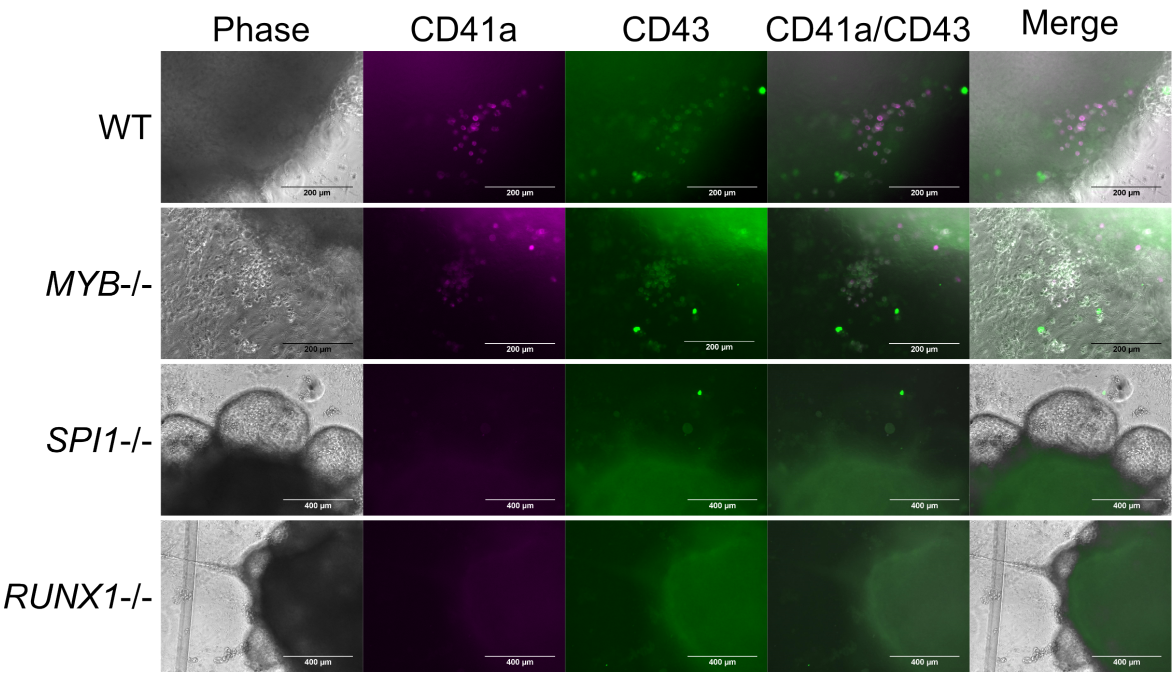

Supplementary figure S5. CD41a$^+$CD43$^+$ early progenitors are detected in WT and MYB-/- EBs. *Related to Figure 4.* WT, *MYB*$^{-/-}$, *RUNX1*$^{-/-}$ and *SPI1*$^{-/-}$ day 14 EBs were stained for CD41a and CD43 hematopoietic markers and imaged on a EVOS FL Auto Cell Imaging System. CD41a$^+$CD43$^+$ early hematopoietic progenitors can be detected in both *MYB*$^{-/-}$ and WT iPSC differentation while they are completley absent in *SPI1*$^{-/-}$ and *RUNX1*$^{-/-}$ iPSC differentation.

Supplemental experimental procedures

Vector construction

The CRISPR-Cas9 vectors used in this study were based on the dual Cas9- and guide RNA (gRNA)-, puromycin-resistance gene-expressing, pSpCas9n(BB)-2A-Puro (pX462) vector (Cong et al., 2013) (gift from Feng Zhang (Addgene plasmid #48141). Cloning was performed as previously described (Cong et al., 2013) using oligonucleotides JB-73 (5' CACCGGCTTGTGGCCACTGCTGGC 3') and JB-74 (5' AAACGCCAGCA-GTGGCCACAAGCC 3') with pX462 to create pX462-gMYBt; oligonucleotides JB-75 (5' CACCGCAGAAGAACAGTCATTTGA 3') and JB-76 (5' AAACTCAAAT-

GACTGTTCTTCTGC 3') with pX462 to create pX462-gMYBb; oligonucleotides JB-81 (5' CACCGCAGTGACCAGAGTGCCATC 3') and JB-82 (5' AAACGATGGCACTCT-GGTCACTGC 3') with pX462 to create pX462-gRUNX1t; oligonucleotides JB-83 (5' CACCGGGCAATGATGAAAACTACT 3') and JB-84 (5' AAACAGTAGT-TTTCATCATTGCCC 3') with pX462 to create pX462-gRUNX1b; oligonucleotides JB-101 (5' CACCGCTGCGGGGGCTGCACGCTC 3') and JB-102 (5' AAACGAGC-GTGCAGCCCCCGCAGC 3') with pX462 to create pX462-gSPI1t; and oligonucleotides JB-103 (5' CACCGCAGCAGCTCTACCGCCACA 3') and JB-104 (5' AAACTG-TGGCGGTAGAGCTGCTGC 3') with pX462 to create pX462-gSPI1b.

Gene editing and single-cell cloning

Knock-out iPSC lines of *MYB*, *RUNX1* and *SPI1* were generated using a dual guide RNA (gRNA)-targeting strategy (Supplementary Figure S1). The location and sequence of the gRNA pairs designed for targeting the *MYB*, *RUNX1* and *SPI1* genes are shown in Supplementary Figure S2. $2x10^6$ feeder-free iPSCs were transfected with two pX462 plasmids in a single-cell suspension by electroporation (Neon®transfection system, Invitrogen), using a 100μL tip with 10μg DNA (5μg top strand pX462-gRNA and 5 μg bottom strand pX462-gRNA). After one pulse of electroporation at 1000 volts and 40 ms pulse width, $1x10^6$ transfected cells were plated onto a matrigel coated 12 well plate in mTeSR1 supplemented with 10 μmol/L Y-27632 without penicillin/streptomycin. After 48h, cells were puromycin selected (0.4 μg/mL; MP Biomedicals) for 48h. Surviving cells were plated at 10^4 cells per 10 cm dish on mitotically-inactivated mouse embryonic fibroblast feeder cells (MEF; outbred Swiss mice established and maintained at the Department of Pathology, Oxford (Chia et al., 2005; Gardner, 1982)) on gelatin-coated tissue culture plates in hESC medium (KO-DMEM, 2 mmol/L L-Glutamine, 100 mmol/L non-essential amino acids, 20% serum replacement, and 8 ng/mL basic fibroblastic growth factor (FGF2)), supplemented with 10 μmol/L Y-27632 on the day of the plating. After 7 days of expansion, individual single-cell colonies were picked manually onto a matrigel coated 96 well plate in mTeSR1. Clones were expanded and pre-screened using high-resolution melt analysis (HRM) on a StepOnePlus Real-Time PCR System

(ThermoFisher). AmpliTaq®Gold DNA Polymerase (ThermoFisher) was used with LCGreen Plus+ (BioChem) melting dye. The following primers were used: JB-71 (5' ACAGGAAGGTTATCTGCAGGAGTCT 3') + JB-72 (5' AGTGGCAGGG-AGTTGAGCTGTA 3') for *MYB*, JB-79 (5' ATCACTACACAAATGCCCTAAAAGTG 3') + JB-80 (5' TTAAATCTTGCAACCTGGTTCTTCA 3') for *RUNX1* and JB-99 (5' CAGACCATTACTGGGACTTCCA 3') + JB-100 (5' GGGTATCGAGGACGTGCATCT 3') for *SPI1*. Genetically modified clones detected by HRM (Supplementary Figure S3A) were sequenced and analysed for insertions and/or deletions, after which, double and single knock-out iPSC clones were expanded and characterized. The first round of transfection produced several single out-of-frame deletion *MYB* clones but did not result in any double out-of-frame knock-out clones. We therefore used a single out-of-frame knock-out clone, clone D6, for re-targeting. Clone D6 was expanded and transfected with the two pX462 Cas9-gRNA-puromycin expressing plasmids targeting *MYB* and processed as previously. After a second round of targeting, several out-of frame knock-outs were generated and 3 clones (Clone B5, C5 and E6) were used in this study. Sequence analysis of the three $MYB^{-/-}$, two $MYB^{+/-}$, single $RUNX1^{-/-}$ and single $SPI1^{-/-}$ iPSC clones are shown in Supplementary Figure S3C. After clonal expansion, presence of wild-type cells in the single-cell clone knock-out iPSC lines was excluded by PCR (Supplementary Figure S3B) The cell clones showed normal undifferentiated morphology, expressed pluripotency markers Tra-1-60 and Nanog and no gross karyotypic abnormalities were detected by SNP array (Supplementary Figure S4).

Supplementary references

Chia, R., Achilli, F., Festing, M.F., Fisher, E.M., 2005. The origins and uses of mouse outbred stocks. Nat Genet 37, 1181–1186.

Cong, L., Ran, F.A., Cox, D., Lin, S., Barretto, R., Habib, N., Hsu, P.D., Wu, X., Jiang, W., Marraffini, L.A., Zhang, F., 2013. Multiplex Genome Engineering Using CRISPR/Cas System. Science. 339, 403–406.

Gardner, R.L., 1982. Investigation of cell lineage and differentiation in the extraembryonic endoderm of the mouse embryo. J. Embryol. Exp. Morphol. 68, 175–198.

Osato, M., 2014. An unsung runt 6e isoform for HSC expansion. Blood 123, 3684–3686.
