# Supplementary material for: Human Induced Pluripotent Stem Cell-Derived Macrophages Share Ontogeny with MYB-Independent Tissue-Resident Macrophages
Source: Stem Cell Reports. 2017 Jan 19;8(2):334–45. doi: 10.1016/j.stemcr.2016.12.020 (PMC5312255; doi:10.1016/j.stemcr.2016.12.020)
Supplement: Document S2. Article plus Supplemental Information [file mmc2.pdf]

Stem Cell Reports
Article

OPEN ACCESS

Human Induced Pluripotent Stem Cell-Derived Macrophages Share Ontogeny with *MYB*-Independent Tissue-Resident Macrophages

Julian Buchrieser,[1,*] William James,[1] and Michael D. Moore[1]
[1]Sir William Dunn School of Pathology, University of Oxford, South Parks Road, Oxford OX1 3RE, UK
*Correspondence: julian.buchrieser@path.ox.ac.uk
http://dx.doi.org/10.1016/j.stemcr.2016.12.020

SUMMARY

Tissue-resident macrophages, such as microglia, Kupffer cells, and Langerhans cells, derive from *Myb*-independent yolk sac (YS) progenitors generated before the emergence of hematopoietic stem cells (HSCs). *Myb*-independent YS-derived resident macrophages self-renew locally, independently of circulating monocytes and HSCs. In contrast, adult blood monocytes, as well as infiltrating, gut, and dermal macrophages, derive from *Myb*-dependent HSCs. These findings are derived from the mouse, using gene knockouts and lineage tracing, but their applicability to human development has not been formally demonstrated. Here, we use human induced pluripotent stem cells (iPSCs) as a tool to model human hematopoietic development. By using a CRISPR-Cas9 knockout strategy, we show that human iPSC-derived monocytes/macrophages develop in an *MYB*-independent, *RUNX1*-, and *SPI1* (*PU.1*)-dependent fashion. This result makes human iPSC-derived macrophages developmentally related to and a good model for *MYB*-independent tissue-resident macrophages, such as alveolar and kidney macrophages, microglia, Kupffer cells, and Langerhans cells.

INTRODUCTION

Adult murine macrophages, in contrast to most other adult hematopoietic cells which renew from hematopoietic stem cells (HSCs) (Gekas et al., 2005), can derive from all three temporally and spatially distinct hematopoietic waves arising in the mouse embryo. A first wave occurs between E7.0 and E7.5 in the blood islands of the yolk sac (YS), producing *Myb*-independent nucleated erythrocytes, megakaryocytes, and macrophages (Mucenski et al., 1991). From E8.25, a second wave of hematopoietic cells emerge in the YS producing erythromyeloid progenitors (EMPs) (Palis et al., 1999, 2001), via a *Runx1*-dependent endothelial to hematopoietic transition (EHT) (Chen et al., 2009), that are capable of monocyte, macrophage, granulocyte, megakaryocyte, and erythrocyte differentiation. The presence of some kit+ EMPs (Schulz et al., 2012) and CD11 b^{high}F4/80^{low} monocytes in the fetal liver of *Myb*$^{-/-}$ mouse embryos (Gomez Perdiguero et al., 2015) suggests that EMP-derived monocytes and macrophages can develop independently of the transcription factor *Myb*. The third wave, from E10.5, consists of HSCs that are generated in the aorto-gonado-mesonephros (AGM) region of the embryo through *Runx1*-dependent EHT (Chen et al., 2009). HSCs depend on the transcription factor *Myb* for their maintenance and self-renewal (Lieu and Reddy, 2009; Schulz et al., 2012). While *Myb* is differentially required for different macrophage populations, primitive erythrocytes are the only cells that still arise in *Runx1*$^{-/-}$ (Okada et al., 1998; Okuda et al., 1996; Wang et al., 1996) or *Spi1*$^{-/-}$ (*Pu.1*) embryos (Scott et al., 1994). Murine YS-derived macrophages and fetal monocytes seed most tissues before HSC-derived definitive hematopoiesis (Ginhoux et al., 2010; Hoeffel et al., 2015; Kierdorf et al., 2013; Gomez Perdiguero et al., 2015; Schulz et al., 2012; Sheng et al., 2015) where, with the exception of dermal (Tamoutounour et al., 2013), gut (Bain et al., 2014), and a fraction of cardiac macrophages (Epelman et al., 2014), they self-renew throughout life with minor contribution from adult blood monocytes (Epelman et al., 2014; Hashimoto et al., 2013).

Our knowledge of human embryonic myeloid development and the ontogeny of tissue-resident macrophages is much more limited due to the difficulty obtaining human embryos at such an early developmental stage (Ivanovs et al., 2011). That said, several human studies are consistent with those in the mouse, in that most resident macrophage populations are independent of adult bone marrow (BM)-derived monocytes. Dermal macrophages remain of recipient origin for a prolonged time after HSC transplantation (Haniffa et al., 2009). After human hand allograft, Langerhans cells in the transplanted limb are still of donor origin 10 years post-transplantation (Kanitakis et al., 2011). Moreover, patients harboring mutations of *GATA-2* or *IRF8* have normal numbers of Langerhans cells and macrophages in the absence of circulating monocytes (Bigley et al., 2011; Bigley and Collin, 2011).

Human embryonic and induced pluripotent stem cells (hESCs/iPSCs) have been extensively studied with the aim of generating true transplantable HSCs with limited success (Ackermann et al., 2015), and although CD14^{+} macrophages can be easily derived from iPSCs (Karlsson et al., 2008; van Wilgenburg et al., 2013), their ontogeny is currently unknown. Klimchenko et al. (2011) have shown that hESC-derived monocytes/macrophages closely resemble, both transcriptionally and functionally, fetal-liver-derived monocytes/macrophages from first-trimester

 © 2017 The Authors.
This is an open access article under the CC BY license (http://creativecommons.org/licenses/by/4.0/).

fetuses. Vanhee et al. (2015) recently showed, using an *MYB*-eGFP reporter hESC line, that $CD14^+$ macrophages were generated through a $CD34^+CD43^+GFP^-$ hematopoietic precursor. Although these observations are suggestive of an *MYB*-independent fetal-like ontogeny of human iPSC-derived monocytes/macrophages, no study has formally investigated the transcription factor requirement of human iPSC myelopoiesis.

Our laboratory has developed a feeder-free embryoid body (EB)-based protocol for the generation of a homogeneous monocyte/macrophage population from hESCs/iPSCs (Karlsson et al., 2008; van Wilgenburg et al., 2013). These cells express classical mononuclear phagocyte markers ($CD14^{high}CD16^{low}CD163^+CD11b^+$), are morphologically similar to blood-derived monocytes/macrophages, and display a macrophage cytokine expression profile while resting or activated using classical (interferon-γ/lipopolysaccharide [LPS]) or alternative activation (interleukin [IL]-4) (van Wilgenburg et al., 2013). In previous studies, macrophages produced using this protocol have been shown to display oxidative burst capacity (Flynn et al., 2015), high phagocytic capacity, and cytokine response on activation and are infectible by HIV-1 (van Wilgenburg et al., 2014), dengue virus (Cowley et al., 2012), influenza virus (van Wilgenburg et al., 2016), *Leishmania* (E. Gluenz, unpublished) and *Mycobacterium tuberculosis* (M. Gutierrez, unpublished). Although these monocytes/macrophages have been extensively studied, mapping their identity onto human hematopoietic development has been hindered by the limited data on human embryos, the lack of definitive human phenotypic markers discriminating tissue-resident macrophages from fetal monocytes or adult blood monocytes, as well as the lack of anatomical location in an iPSC system; thus we set out to genetically define their ontogeny.

With the aim of studying the requirement of *MYB*, *RUNX1*, and *SPI1* in the in vitro differentiation of these monocytes/macrophages from human iPSCs and mapping human hematopoiesis onto that of the mouse, we established knockout iPSC lines for each of these genes using the CRISPR-Cas9 system. We show that the monocytes/macrophages produced are *MYB* independent but *RUNX1* and *SPI1* dependent, which would tie them to *MYB*-independent myelopoiesis in the YS.

RESULTS

iPSC-Derived Monocyte/Macrophage Development Is *MYB* Independent but *RUNX1* and *SPI1* Dependent

We established knockout iPSC lines of *MYB*, *RUNX1*, and *SPI1* using a dual-guide RNA (gRNA)-targeting strategy (Figures S1–S4). To investigate the capacity of $MYB^{-/-}$, $MYB^{-/+}$, $SPI1^{-/-}$, and $RUNX1^{-/-}$ iPSCs to undergo myelopoiesis, we differentiated the iPSCs to monocytes/macrophages using our EB differentiation protocol (Karlsson et al., 2008; van Wilgenburg et al., 2013). Over a period of 30 days, wild-type (WT) and $MYB^{-/+}$ iPSCs produced an average of 3×10^6 monocytes/macrophages per well containing eight EBs, suggesting *MYB* haploinsufficiency had no effect on monocyte/macrophage commitment (Figure 1A). Interestingly, $MYB^{-/-}$ iPSCs were capable of myeloid differentiation and produced 2-fold more $CD14^+$ cells than WT and $MYB^{-/+}$ (Figure 1A). When plotted as a noncumulative production of $CD14^+$ monocytes/macrophages over time, it is apparent that $MYB^{-/-}$ iPSCs produce significantly more monocytes/macrophages than the WT control or $MYB^{-/+}$ iPSCs during the first weeks of production (Figure 1B). In contrast, $SPI1^{-/-}$ and $RUNX1^{-/-}$ iPSCs were unable to produce any $CD14^+$ monocytes/macrophages (Figure 1A), although the EBs increased in size as expected and were comparable in their morphology when compared with WT or $MYB^{-/-}$ EBs (Figures 1C and 1D).

$MYB^{-/-}$ iPSC-Derived Monocytes/Macrophages Display No Major Phenotypic or Functional Defects and Show a Similar Tissue-Resident Transcriptional Signature to WT Cells

As *MYB* is a major player in hematopoietic differentiation and hematopoietic cell function, we checked that the deletion of *MYB* did not affect the phenotype or function of the monocytes/macrophages generated. $MYB^{-/-}$ and WT monocytes/macrophages showed no difference in morphology (eosin and methylene blue staining), phenotype (classical mononuclear phagocyte markers CD45, CD11b, CD14, and CD16), reactive oxygen species (ROS) production, tumor necrosis factor (TNF)-α release after LPS stimulation, and phagocytosis (zymosan uptake) (Figures 2A–2E, 2G, and 2H). While *MYB* knockout might affect macrophage function in a subtle way, $MYB^{-/-}$ and WT monocytes/macrophages are overall highly similar. To ensure that in the absence of *MYB* the monocytes/macrophages produced are from the same developmental pathway as the WT cells, we analyzed the expression of *MAF*, *CSFR1*, *FLT3*, and *CCR2*, which are the most differentially expressed in mouse phenotypically YS-derived ($CD11b^{low}F4/80^{high}$) and blood-derived ($F4/80^{low}$ $CD11b^{high}$) macrophages (Schulz et al., 2012). Relative expression of *MAF*, *CSFR1*, *FLT3*, and *CCR2* was quantified by RT-qPCR in primary blood monocytes and freshly harvested WT or $MYB^{-/-}$ iPSC-derived monocytes/macrophages (Figures 3A–3D). *CSFR1* and *MAF* were expressed at significantly higher levels in iPSC-derived monocytes/macrophages than primary blood monocytes, while *FLT3* and *CCR2* were undetected in WT or $MYB^{-/-}$ iPSC-derived monocytes/macrophages but were expressed in primary

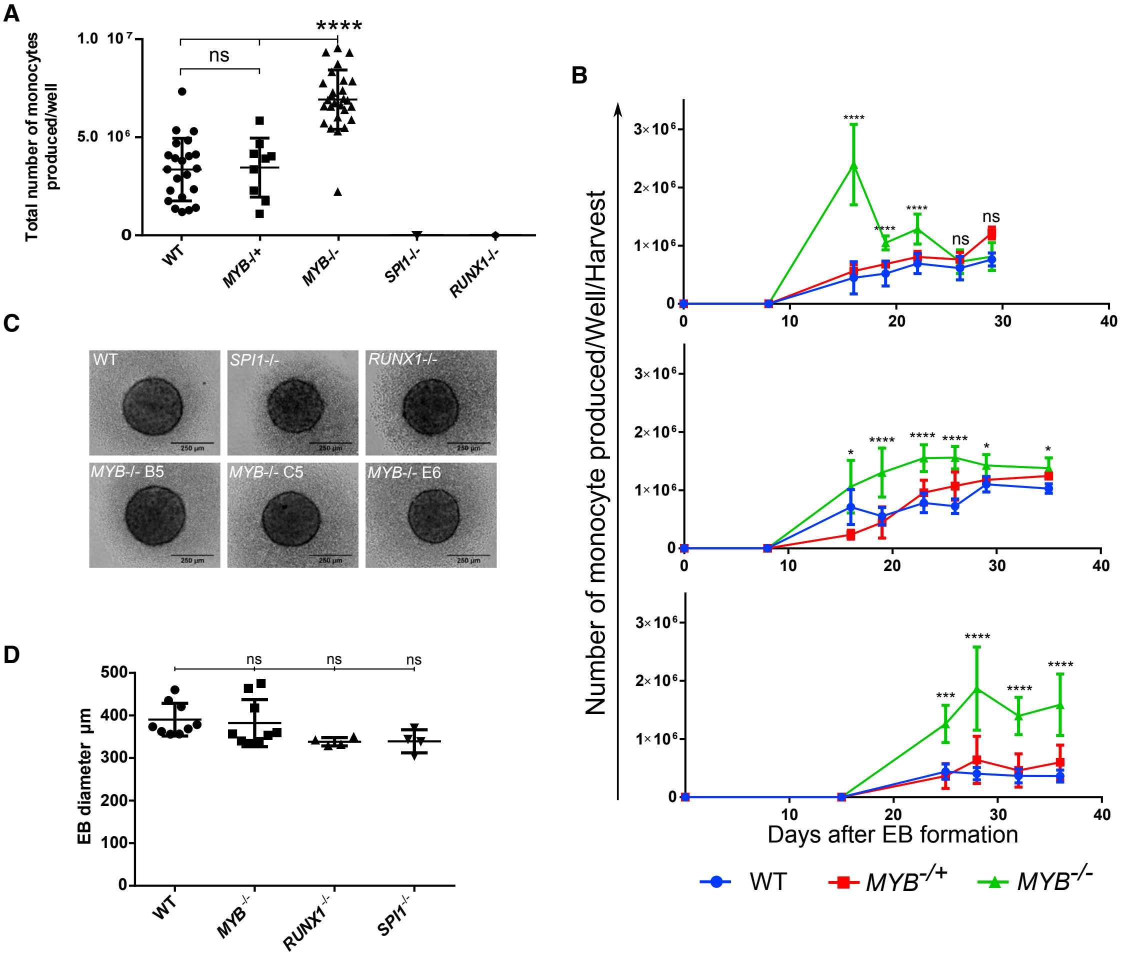

Figure 1. Monocyte/Macrophage Production Capacity of WT, *MYB*$^{-/+}$, *MYB*$^{-/-}$, *RUNX*$^{-/-}$, and *SPI1*$^{-/-}$ iPSCs

(A) Total number of $CD14^+$ cells produced per well containing eight EBs over a period of 30 days, plotted with mean and SD, three independent experiments, number of total wells: WT n = 22 (from three independent clones), *MYB*$^{-/-}$ n = 27 (from three clones), *MYB*$^{-/+}$ n = 9 (from one clone), *SPI1*$^{-/-}$ n = 9 (from one clone), and *RUNX1*$^{-/-}$ n = 9 (from one clone). Cell counts have been normalized to the $CD14^+$ percentage of each replica (over 90% of the cells produced were $CD14^+$ for each well independently of genetic modifications). Statistical comparisons were performed using a nonparametric Mann-Whitney test, ****$p < 0.0001$.

(B) Noncumulative production of monocytes per well over a period of 30 days of the three independent experiments shown in (A). Each time point represents the mean number of $CD14^+$ cells harvested per well of *MYB*$^{-/-}$ (n = 9), WT (n = 6), and *MYB*$^{-/+}$ (n = 3) iPSCs. Error bars denote SD. Statistical comparisons were done using a two-way ANOVA, ns, nonsignificant, *$p < 0.05$, ***$p < 0.001$, ****$p < 0.0001$.

(C) Representative image of WT, *MYB*$^{-/-}$, *MYB*$^{-/+}$, *RUNX1*$^{-/-}$, and *SPI1*$^{-/-}$ EBs after 1 day of differentiation.

(D) Mean diameter with SD of WT, *MYB*$^{-/-}$, *RUNX1*$^{-/-}$, and *SPI1*$^{-/-}$ EBs; each data point represents the mean diameter of one independent experiment (n = 16). Diameter was calculated using ImageJ, and statistical comparisons were performed using a nonparametric one-way ANOVA comparing the mean of each column with the mean of the WT control column.

blood monocytes. These observations tie in with the mouse observation that *MAF* (involved in macrophage proliferation; Aziz et al., 2009) and *CSFR1* (macrophage colony stimulating factor [M-CSF] receptor) are highly expressed on YS-derived tissue-resident macrophages, while *FLT3* (expressed in pluripotent hematopoietic progenitors) and the chemokine receptor *CCR2* is low. A 2-fold increase of *CSFR1* was observed in *MYB*$^{-/-}$ monocytes/macrophages, but this increase is comparatively small compared with the 10-fold difference with primary blood monocytes.

MYB Knockout Results in Increased Number of Hematopoietic Progenitors within the EB

YS macrophages and fetal monocytes have a high-proliferative capacity compared with BM-derived monocytes (van de Laar et al., 2016), thus we set out to identify whether the

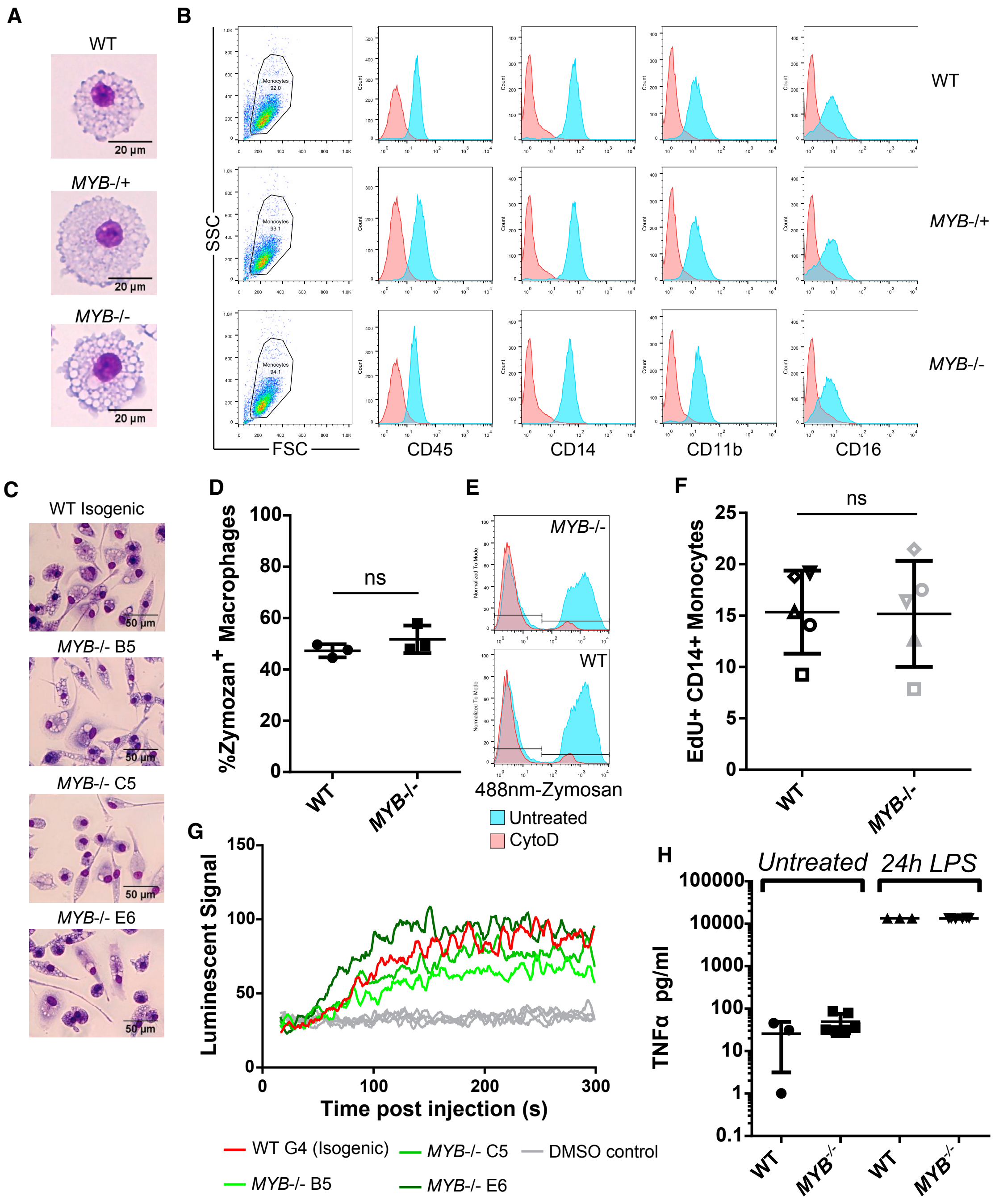

Figure 2. *MYB*$^{-/-}$ Monocytes/Macrophages Display No Phenotypic or Functional Differences Compared with WT Control

(A) Representative images of eosin and methylene blue stain of cytospined WT, *MYB*$^{-/+}$, and *MYB*$^{-/-}$ monocytes/macrophages on the day of harvest.

(B) Flow cytometry staining of monocytes/macrophages for common myeloid cell surface makers, showing live cell gate on the left and histogram plots on the right, antibody staining (blue) and isotype (red).

(C) Representative images of eosin and methylene blue stain of WT, *MYB*$^{-/+}$, and *MYB*$^{-/-}$ monocytes/macrophages differentiated for 7 days in M-CSF.

(D) Phagocytosis assay measuring the fluorescently labeled zymosan uptake by 1 week differentiated WT and *MYB*$^{-/-}$ macrophages. Cells were analyzed by flow cytometry and the percentage of AlexaFluor-488-zymosan^{+} macrophages was plotted. The experiment was run in triplicate, and statistical comparisons were done using an unpaired t test. Error bars denote SD between replicates.

(legend continued on next page)

increase in monocytes/macrophages from the $MYB^{-/-}$ iPSC lines was due to the proliferation of the monocytes/macrophages during the first week of production (a time point at which we have observed the maximum rate of cell division; data not shown). To detect the rate of monocyte/macrophage division, we pulsed the monocytes with 5-ethynyl-2′-deoxyuridine (EdU) for 2 hr (Figure 2F). No significant difference was observed in monocytes/macrophages undergoing S phase ($CD14^{+}EdU^{+}$) between $MYB^{-/-}$ and WT, suggesting that the increased monocyte/macrophage production is most likely due to an event upstream of the monocyte/macrophage differentiation stage.

To investigate whether $MYB^{-/-}$ EBs generated more hematopoietic progenitors, we enzymatically dissociated EBs at various time points into single cells and stained for expression of the pan-hematopoietic marker CD45 and the endothelial/hematopoietic marker CD34. Hemogenic and non-hemogenic endothelial cells are labeled $CD34^{+}CD45^{-}$, hematopoietic progenitor cells are labeled $CD34^{+}CD45^{+}$, and committed hematopoietic cells are labeled $CD34^{-}CD45^{+}$ (Figure 4A). While the percentage of total $CD34^{+}$ cells was similar between WT and $MYB^{-/-}$ on days 9 and 11, very few $CD34^{+}CD45^{+}$ cells were detected in the WT condition, whereas a significant population of $CD34^{+}CD45^{+}$ cells (close to 20% of all single- and double-stained cells) was present in $MYB^{-/-}$ (Figures 4B and 4C). This would suggest that $MYB^{-/-}$ iPSCs undergo hematopoietic commitment earlier than WT iPSCs. Day 14 WT EBs show a major hematopoietic commitment, $CD45^{+}CD34^{-}$ representing 63% of the single- and double-stained population, while $CD34^{+}$ $CD45^{+}$ cells represent only 1.4% of the total (Figures 4B and 4C), indicating that the hematopoietic progenitor pool is small within the WT EBs. $MYB^{-/-}$ EBs, on the other hand, display two distinct hematopoietic sub-populations at day 14; of the single- or double-stained cells, 19.5% were $CD34^{+}$ $CD45^{+}$ and 47% were $CD45^{+}$ $CD34^{-}$, indicating that a significantly larger hematopoietic progenitor cell pool is maintained within the $MYB^{-/-}$ EBs. To further investigate the progenitors present at day 14 of differentiation, we stained EBs for CD41a and CD43, two markers present on lineage-restricted hematopoietic progenitors derived from an early EHT (Rafii et al., 2013) (Figure S5). $CD41a^{+}CD43^{+}$ hematopoietic progenitors can be detected in WT and $MYB^{-/-}$ iPSC differentiation; they are absent from $RUNX1^{-/-}$ and $SPI1^{-/-}$ differentiations.

iPSC-Derived Erythrocytes and Granulocytes Are Dependent on *MYB*, *RUNX1*, and *SPI1*

To investigate the progenitor potential of $MYB^{-/-}$, $RUNX1^{-/-}$, and $SPI1^{-/-}$ iPSCs, EBs were differentiated for 14 days as for monocyte/macrophage differentiation, followed by enzymatic dissociation into single cells. The day 14 time point was chosen, as earlier time points (day 9 or day 11) derived only a small number of hematopoietic colonies. Single-cell suspensions were plated into MethoCult H4434, which supports the growth of erythroid progenitors (colony-forming unit [CFU]-E and burst forming unit [BFU]-E), granulocyte-macrophage progenitors (CFU-GM, CFU-G, and CFU-M), and multi-potential granulocyte, erythroid, macrophage, and megakaryocyte progenitors (CFU-GEMM). After 14 days of expansion, colonies were scored according to morphology. WT iPSCs generated CFU-E, CFU-GM, and CFU-M colonies, whereas $MYB^{-/-}$ iPSCs generated only CFU-M colonies (Figures 4D and 4E). $RUNX1^{-/-}$ and $SPI1^{-/-}$ iPSC were unable to generate any hematopoietic colonies (data not shown).

DISCUSSION

In this report, we have used CRISPR-Cas9 to knock out key transcription factors in human iPSCs that are known to be involved in murine myeloid development. We used a feeder-free EB differentiation model to understand the developmental ontology and transcription factor requirement of in vitro generated monocytes/macrophages and thus map human iPSC myelopoiesis onto mouse hematopoietic development. Our results show that, using this

(E) Representative histograms of WT and $MYB^{-/-}$ iPSC-derived monocyte/macrophages of AlexaFluor-488-zymosan uptake, untreated (blue) and cytochalasin D-treated negative control (red).
(F) First harvest monocytes/macrophages pulsed with EdU and $CD14^{+}EdU^{+}$ cells that had undergone DNA replication were detected by flow cytometry after Click-iT staining. Five independent experiments are shown with mean and SD (symbols represent paired experiments), where $MYB^{-/-}$ represents the pooled results obtained for three different $MYB^{-/-}$ iPSC clones and WT the pooled result obtained from technical triplicates from a single clone. Statistical comparisons were done using a paired t test.
(G) Monocytes/macrophages from WT and $MYB^{-/-}$ iPSCs were treated with luminol reagent in the presence or absence of PMA stimulation. Individual wells were monitored for light released at 1 s intervals for 300 s in triplicate using a PHERAstar FS (BMG Labtech). The mean of three wells was normalized to the average of the 5 s before luminol addition and are plotted with smoothing for clarity (average of six neighboring data points).
(H) ELISA measuring the TNF-α released by 1 week differentiated macrophages after 24 hr LPS stimulation compared with untreated controls. Results are plotted with mean and SD. No significant difference was observed using one-way ANOVA between WT and $MYB^{-/-}$ cells.

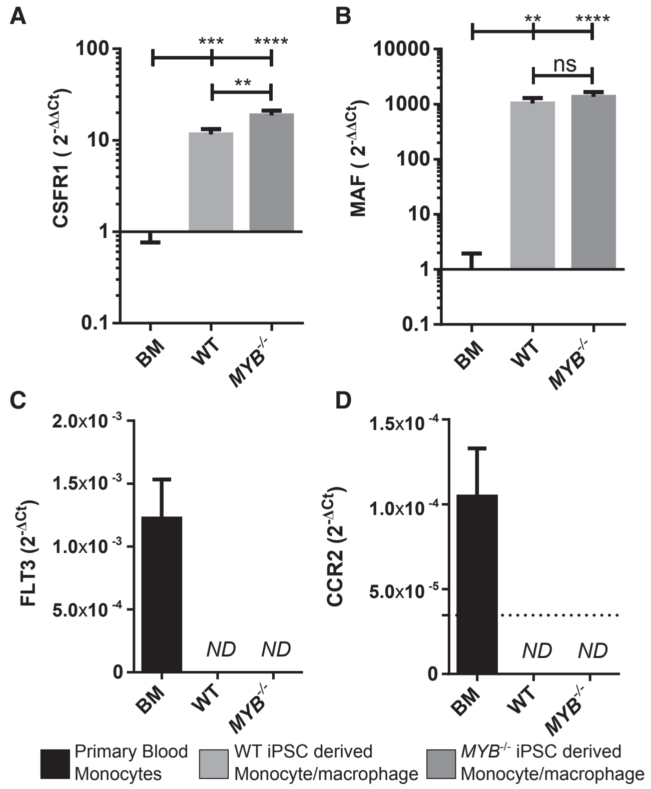

Figure 3. iPSC-Derived Monocytes/Macrophages Display a Similar mRNA Expression to Mouse *MYB*-Independent Macrophages

Relative expression of (A) *CSFR1*, (B) *MAF*, (C) *FLT3*, (D) *CCR2* mRNA in primary blood monocytes (3 donors, n = 3), WT iPSC-derived monocytes/macrophages (three independent differentiations, n = 3), and *MYB*$^{-/-}$ iPSC-derived monocytes/macrophages (two independent differentiations, three clones, n = 6). (A and B) Expression data were normalized to *EF1α* endogenous control, and relative mRNA expression to blood monocytes ($2^{-\Delta\Delta CT}$) is plotted. (C and D) C_T values of *CCR2* and *FLT3* were undetectable in all iPSC samples; therefore, the relative quantity of mRNA normalized to *EF1α* endogenous control is plotted ($2^{-\Delta CT}$). The dotted line represents the limit of detection equal to the $2^{-\Delta CT}$ of the no-template control. Error bars denote SD. Statistical comparisons were done using a one-way ANOVA for each gene. ns, nonsignificant; $^{**}p < 0.01$, $^{***}p < 0.001$, $^{****}p < 0.0001$. ND, non-detected/equal to no-template control.

protocol, iPSC-derived monocytes/macrophages are independent of the transcription factor *MYB*, which is required for definitive AGM hematopoiesis in the mouse (Mucenski et al., 1991; Mukouyama et al., 1999; Sumner et al., 2000), while being dependent on the transcription factor *RUNX1*, which is required for EHT (Chen et al., 2009), as well as *SPI1*, which is required for myeloid differentiation and plays a major role in many monocyte and macrophage functions (Anderson et al., 1998; Zakrzewska et al., 2010). The hematopoietic progenitors within the EBs are capable of macrophage, granulocyte, and erythrocyte potential, but in the absence of *MYB*, only macrophage colonies are detected by colony-forming assay in semi-solid media, suggesting that both CFU-E and CFU-GM are generated in an *MYB*-dependent fashion from EMPs. Furthermore, loss of erythrocyte colony potential in the *MYB* knockout iPSCs would indicate the absence of *RUNX1*- and *MYB*-independent primitive erythrocyte precursors within the EBs using this protocol. Taken together, our results suggest that iPSC-derived monocytes/macrophages can derive independently of *MYB* from *RUNX1*- and *SPI1*-dependent EMPs (Figure 5). The presence of primitive unilineage macrophage progenitors cannot be excluded without proper clonal analysis. Further investigation at the clonal level of the mesodermal, endothelial, and hematopoietic progenitors will be critical to define the relative contribution of monopotent primitive macrophage progenitors and EMPs to *MYB*-independent iPSC monocytes/macrophages.

While we cannot exclude the possibility that some *MYB*-dependent hematopoietic progenitor cells (HPCs)/HSCs are generated during normal WT iPSC differentiation, we would expect a reduction in monocyte/macrophage production if the majority of monocytes/macrophages were derived from *MYB*-dependent progenitors. In contrast, we observed an increase in production of monocytes/macrophages in the *MYB*$^{-/-}$ iPSCs, without any major change in phenotype or function of the cells. Furthermore, using a similar EB-based human embryonic stem cell differentiation protocol, Vanhee et al. (2015) observed that multipotent HPCs expressing high levels of *MYB* were not generated in their cultures and that macrophages were generated from precursors not expressing detectable *MYB*. Combined with our data, this strongly suggests that in WT iPSC differentiation, most, if not all, monocytes/macrophages are produced in an *MYB*-independent fashion, and the contribution of *MYB*-dependent multilineage HPC/HSC-derived hematopoiesis in our EB-based monocyte differentiation protocol is negligible.

Interestingly, in addition to the increased monocyte/macrophage production, we observed an increased number of $CD34^+$ $CD45^+$ HPCs within *MYB*$^{-/-}$ EBs. A very similar phenotype has been observed in mouse *Myb*$^{-/-}$ ESC differentiation by Clarke et al. (2000). First, they observed that *Myb*$^{-/-}$ ESCs were capable of macrophage and primitive erythrocyte colony formation, but the kinetics of formation was different between the control line and the *Myb*$^{-/-}$ ESCs. The number of CFU-E was lower in *Myb*$^{-/-}$ while the generation of CFU-M was increased at day 7 when compared with the WT control. Second, no BFU-E were generated by *Myb*$^{-/-}$ ESCs, indicating a block to definitive erythrocyte production. Third, they observed a higher number of $CD34^+$Sca-1^+ HPCs within the *Myb*$^{-/-}$ EBs, but these progenitors were unable to progress further in differentiation. This could be due to an increased hematopoietic

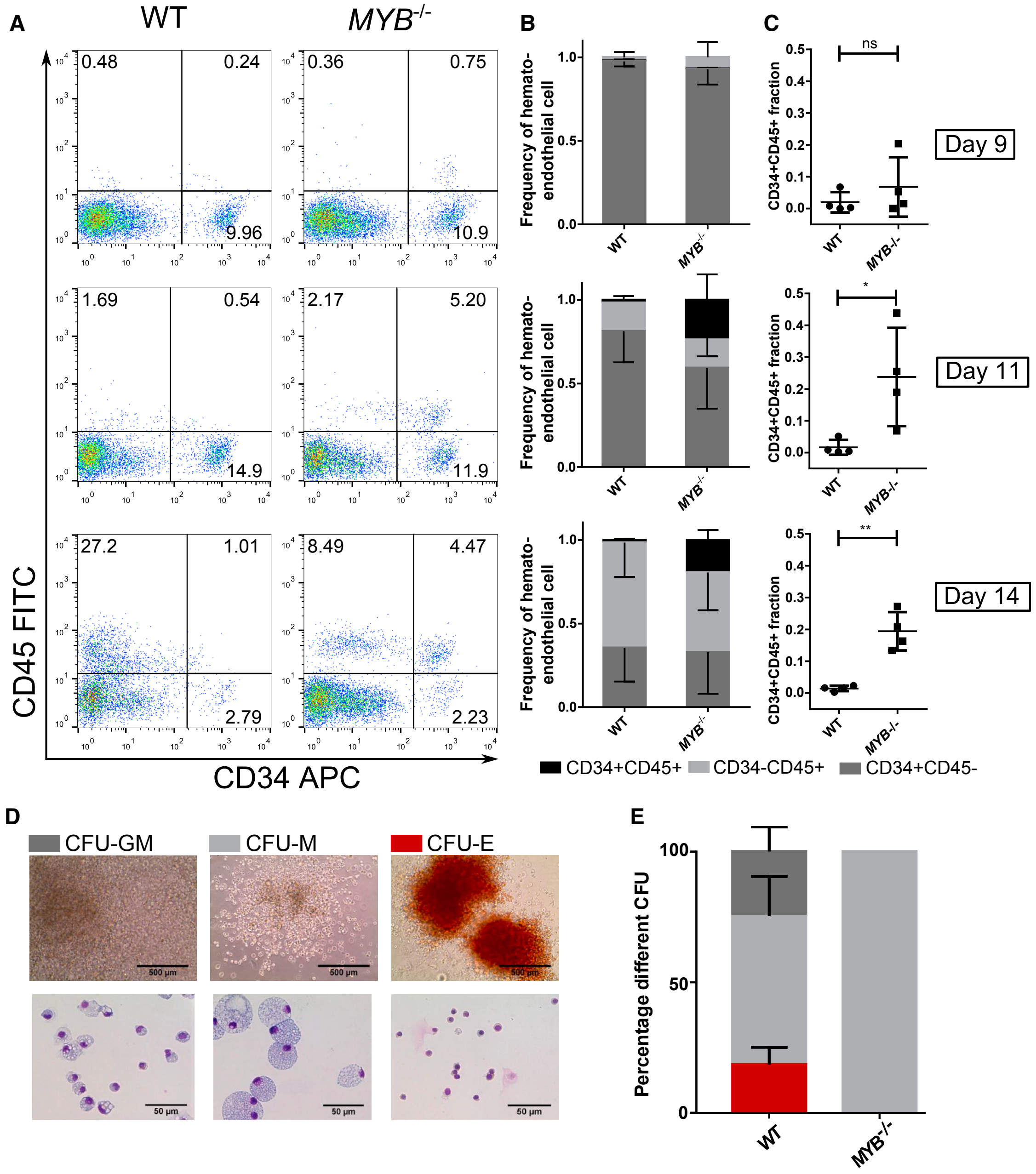

Figure 4. Study of the Progenitor Cells within the iPSC-Derived EBs

(A) Representative plots of day 9, 11, and 14 EBs of WT and *MYB*$^{-/-}$ iPSCs, which were enzymatically dissociated, stained for expression of CD34 and CD45, and analyzed by flow cytometry. Hemogenic and nonhemogenic-endothelial cells are CD34^{+}CD45^{-}, HPCs are CD34^{+}CD45^{+}, and differentiated hematopoietic cells are CD34^{-}CD45^{+}.

(B) Relative proportion of CD34^{+}CD45^{+}, CD34^{+}CD45^{-}, and CD34^{-}CD45^{+} populations on day 9, 11, and 14 normalized to the total number of single- and double-positive cells. Mean and SD of four repeats are plotted.

(C) Relative proportion of CD34^{+}CD45^{+} populations on day 9, 11, and 14. Statistical comparisons were done using a paired t test. ns, nonsignificant; *$p < 0.05$, **$p < 0.01$.

(D) Image of the different colony types. The images on the left show a bright-field image of representative CFU-E, CFU-M, and CFU-GM WT colonies in methylcellulose media at day 14, while the images on the right show the cytospined and eosin and methylene blue-stained cells present within the three different types of colony.

(legend continued on next page)

commitment, progenitor proliferation, or an accumulation of committed erythrocyte/granulocyte progenitors that cannot progress further through differentiation due to the lack of *Myb*. It will be interesting to understand the precise mechanism of action underlying this increase in precursor cells and monocytes/macrophages.

With the mounting data suggesting that tissue-resident macrophages and BM monocyte-derived macrophages can play different roles in diseases such as cancer (Lahmar et al., 2015) and parasite infection (Rückerl and Allen, 2014), having access to authentic embryonic-derived monocytes and macrophages in vitro will be of considerable scientific value. Patient-derived tissue-resident macrophages are very difficult to obtain, are inherently genetically variable, and are notoriously difficult to genetically modify, making their study laborious and unreliable. On the other hand, iPSCs can be generated from a patient with a specific genetic background and can be modified by multiple mechanisms, such as lentiviral transduction or CRISPR-Cas9 gene editing. The demonstration that *MYB*-independent monocytes/macrophages can be generated in our differentiation protocol lays the foundation for their use in the development of reliable protocols for generating the tissue-specific subtypes of macrophages for the in vitro study of their role in pathology and homeostasis. Moreover, iPSC differentiation is a potential source of tissue-resident macrophages for cell therapy, as has recently been shown in the mouse with the use of murine pluripotent stem cell-derived $Myb^{-/-}$ alveolar-like macrophages as a cell source for treating a mouse model of adenosine deaminase deficiency ($ADA^{-/-}$) (Litvack et al., 2016).

EXPERIMENTAL PROCEDURES

Human iPSC Culture

The human iPSC line AH016-03 was the target line for all gene editing in this study. The derivation and characterization of iPSC line AH016-03 has been previously published (Sandor et al., 2017), and was derived from a normal healthy donor, having given signed informed consent, which included derivation of human iPSC lines from skin biopsies (Ethics Committee: National Health Service, Health Research Authority, NRES Committee South Central, Berkshire, UK (REC 10/H0505/71)). The SNP datasets and the Illumina HT12v4 transcriptome array results of the parental cell line have been deposited in Gene Expression Omnibus under accession number GEO: GSM2055806. The SNP datasets of the genetically modified lines have been deposited in GEO: GSE93285. iPSCs were cultured in feeder-free conditions in mTeSR1 (STEMCELL Technologies) on Matrigel (Scientific Laboratory Supplies 354277). Cells were passaged with TrypLE Express (Gibco by Life Technologies) and plated in media containing 10 μmol/L Rho-kinase inhibitor Y-27632 (Abcam). The number of passages was kept to a minimum to reduce the likelihood of genetic change, and cells were frozen in SNP quality-controlled batches from which cells would be thawed for each experiment, to ensure consistency.

EB Formation

Spin-EBs were formed using a 96-well ultra-low adherence plate (Costar 7007). iPSCs were washed with PBS and harvested by incubating the cells for 5 min at 37°C with 1 mL of warm TrypLE Express (Gibco by Life Technologies). Cells were counted, washed with PBS, and resuspended at a final concentration of 1.25×10^5 cells/mL in EB media: mTeSR1 (STEMCELL Technologies), 50 ng/mL BMP-4 (GIBCO- PHC9534), 20 ng/mL stem cell factor (Miltenyi Biotec), 50 ng/mL vascular endothelial growth factor (GIBCO- PHC9394); 100 μL of cell suspension in EB media supplemented with 10 μmol/L Y-27632 was added per well, and the 96-well plate was centrifuged at 100 × *g* for 3 min and incubated for 4 days. EBs were fed at days 1 and 2 by aspirating 50 μL of medium and adding 50 μL of fresh EB medium.

Myeloid Differentiation

After 4 days of EB differentiation, EBs were transferred into a six-well tissue-culture plate (8 EBs/well) (CorningCostar) and resuspended in monocyte/macrophage differentiation media consisting of X-VIVO-15 (Lonza), supplemented with 100 ng/mL M-CSF (Invitrogen), 25 ng/mL IL-3 (R&D), 2 mM glutamax (Invitrogen), 100 U/mL penicillin and 100 mg/μL streptomycin (Invitrogen), and 0.055 mM β-mercaptoethanol (Invitrogen). Two-thirds of the media was changed every 5 days. After the first production of iPSC-derived monocytes/macrophages, non-adherent monocytes/macrophages were harvested from the supernatant every week for staining and counting.

Cell Count and Viability

Cells were counted using NC-3000 Viability and Cell Count Assays (Chemometec) according to the manufacturer's instructions.

EdU Staining

Monocytes/macrophages were harvested and counted, and 1×10^5 cells were transferred into a well of a 12-well plate without media change. Cells were pulsed with 10 μM EdU for 2 hr after which they were harvested and stained according to the manufacturer's protocol for the Click-iT Plus EdU Flow Cytometry Assay Kits (Thermo Fisher).

Eosin and Methylene Blue Staining

Freshly harvested monocyte (5×10^4) cells were either centrifuged at 400 × *g* for 4 min onto a glass slide using a Cytospin 3 (Shandon) or further differentiated for 7 days on glass coverslips

(E) Dissociated day 14 EBs were plated into H4434 MethoCult; after 14 days, colonies were scored. The percentage of each type of colony is displayed as mean with SD (WT n = 5, $MYB^{-/-}$ n = 3 for each knockout clone). Presence of erythroid (CFU-E), granulocyte-macrophage (CFU-GM), and macrophage progenitors (CFU-M) can be detected in WT iPSC differentiation, whereas $MYB^{-/-}$ iPSCs display only CFU-M potential. $RUNX1^{-/-}$ and $SPI1^{-/-}$ iPSCs did not generate any hematopoietic colonies. See also Figure S5.

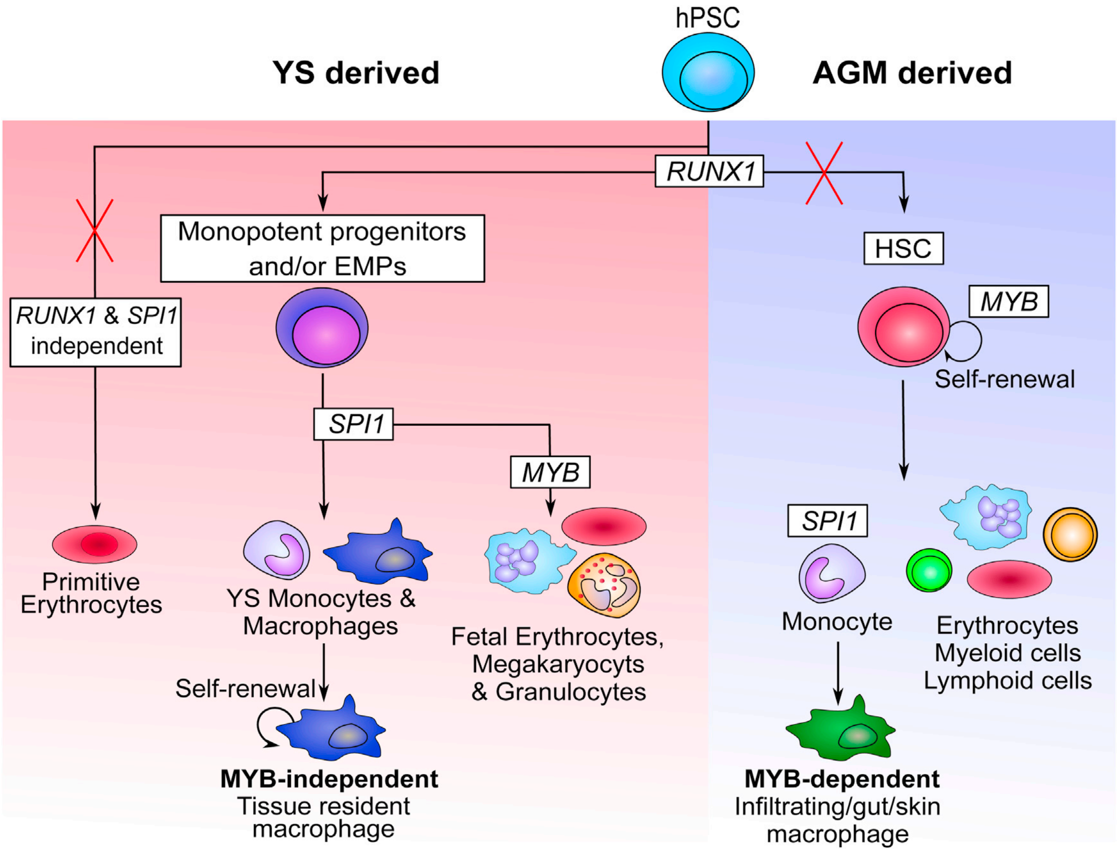

Figure 5. Schematic Representation of the Proposed Model of Lineage Commitment Occurring during Human iPSC Myelopoiesis

In our protocol, when *RUNX1* is knocked out, no hematopoietic cells are observed, including primitive erythrocytes. This suggests the absence of unilineage primitive erythrocytes in this specific differentiation protocol (red cross indicates lack of production in our system) and confirms the mouse observation that *RUNX1* is required for all macrophage lineages. In contrast, *MYB* knockout only blocked erythroid and granulocyte colony-forming potential, which fits with the observation in the mouse that definitive erythrocytes and granulocytes require a functional *MYB* gene. *MYB* knockout does not block monocyte/macrophage differentiation, indicating that most iPSC-derived monocytes/macrophages generated in this study are *MYB* independent. The precursor of these monocytes/macrophages is still unknown, as macrophages could differentiate in a *MYB*-independent fashion from either unilineage primitive macrophage progenitors or EMPs. Furthermore, the fact that *MYB* is not required for myeloid differentiation in this EB differentiation protocol suggests the absence of definitive HSC commitment (red cross indicates lack of production in our system). Taken together, our results fit with the current observation in the mouse and suggest that *MYB*, *RUNX1*, and *SPI1* play a very similar role in mouse and human myeloid development.

in macrophage differentiation media consisting of monocyte/macrophage differentiation media without IL-3 (X-VIVO-15 [Lonza], supplemented with 100 ng/mL M-CSF [Invitrogen], 2 mM glutamax [Invitrogen], 100 U/mL penicillin and 100 mg/μL streptomycin [Invitrogen], and 0.055 mM β-mercaptoethanol [Invitrogen]). Slides were air dried briefly and stained using an eosin and methylene blue staining kit (HemaGurr) as specified by the manufacturer's protocol. Images were acquired using an EVOS inverted microscope.

EB Dissociation and Colony-Forming Assay

Twenty-four EBs per condition were harvested using a 100-μm cell strainer, washed in PBS, and EBs were resuspended in 500 μL of Accumax Solution (Sigma). EBs were incubated for 5 min at 37°C, mechanical dissociation was performed by carefully pipetting up and down using a 200 μL pipette for 1 min, after which the EBs were incubated an extra 5 min at 37°C followed by mechanical dissociation for an additional minute. The resulting cell suspension was filtered using a 70 μm cell strainer before being centrifuged for 5 min at 400 × *g*. Colony-forming cell assay was performed according to the manufacturer's protocol using a cell concentration of 3×10^5 cells/mL resulting in a final plating concentration of 3×10^4 cells per 35 mm dish of MethoCult H4434 (STEMCELL Technologies). Colonies were scored by morphology 14 days after plating.

Flow Cytometry Staining and Antibodies

Harvested monocytes/macrophages or single-cell suspension obtained from EB dissociation were pelleted at 400 × *g* for 5 min and washed once with PBS before being resuspended in 100 μL of fluorescence-activated cell sorting (FACS) buffer (PBS +10 μg/mL

human serum IgG + 1% fetal bovine serum [FBS]). Cells were stained in FACS buffer + antibody (dilution 1:100) for 45 min at 4°C. Cells were then washed using PBS and resuspended in 2% formaldehyde before being analyzed using a FACSCalibur flow cytometer (BD Biosciences). The following antibodies have been used in this study: α-CD14-APC antibody (MEM-15; Immunotools; 21279146), IgG1-APC isotype (PPV-06; Immunotools; 21275516X2), α-CD34-APC (4H11; eBioscience; 17-0349-42), mouse IgG1κ-APC isotype (P3.6.2.8.1; eBioscience; 17-4714-41), α-CD45-fluorescein isothiocyanate (FITC) (MEM-28; ImmunoTools; 21270453X2), mouse IgG1-FITC isotype (PPV-06; ImmunoTools; 21275513X2), α-CD16-APC (LNK16; Immunotools; 21279166X2), α-CD11b-APC (ICRF44; BioLegend; 301309), and mouse IgG1-APC isotype (MOPC-21; BioLegend; 400119).

Phagocytosis Assay

Harvested monocytes/macrophages were plated on tissue-culture plates in macrophage differentiation media for 7 days. On the day of the analysis, negative control wells were pretreated with 10 μM cytochalasin D for 1 hr. After pretreatment, all wells were fed with media containing two particles per cell of 488-zymosan (Z-23373, Thermo Fisher) and incubated for 30 min at 37°C. Macrophages were detached using 5 mM EDTA and 12 mM lidocaine (Sigma) in PBS and membrane-bound zymosan was quenched using 250 μg/mL trypan blue (Gibco) for 5 min before fixation in 2% formaldehyde. Stained cells were analyzed on a FACSCalibur flow cytometer (BD Biosciences).

TNF-α ELISA

Harvested monocytes/macrophages were plated on tissue-culture plates in macrophage differentiation media for 7 days. Positive control wells were activated with LPS at 100 ng/mL for 24 hr. Cleared cell culture supernatants were diluted 1:5 and probed using the TNF-α ELISA Ready set go (88-7346-86, eBioscience) according to the manufacturer's instructions.

ROS Assay

Harvested monocytes were plated onto tissue-culture plates for 24 hr in macrophage differentiation media for 7 days, before analysis for ROS production. The luminol assay was used to identify ROS activity from macrophages, as previously described (Jiang et al., 2012), after stimulation with 200 ng/mL phorbol 12-myristate 13-acetate (PMA). Individual wells were monitored for light released at 1-s intervals for 300 s using a PHERAstar FS (BMG Labtech).

RNA Extraction, Reverse Transcription, and qPCR

Peripheral blood of three healthy adult volunteers was collected according to University of Oxford OHS policy document 1/03, with signed informed consent. Peripheral blood mononuclear cells were isolated from blood by density gradient centrifugation with Ficoll-Paque PLUS (17-1440-03, GE Healthcare) and monocytes were sorted using CD14 MACS beads (130-050-201, Miltenyi). A total of 2×10^6 iPSCs, freshly harvested iPSC-derived monocytes/macrophages, or primary blood monocytes were lysed using RLT buffer (QIAGEN) supplemented with 10 μL of β-mercaptoethanol. RNA extraction was performed using the RNeasy kit (QIAGEN) according to the manufacturer's protocol. Potential DNA contamination was removed by adding a step of Ambion TURBO DNA-free according to the manufacturer's protocol (Life Technologies). Reverse transcription was performed using the RetroScript (Ambion) kit or the high-capacity RNA-to-cDNA kit (Applied Biosystems) according to the manufacturer's protocol. qPCR was performed using Brilliant III SYBR (Agilent) on the Applied Biosystems StepOnePlus Real-Time PCR System. The following primers were used: EF1α forward (5′-CTG AAC CAT CCA GGC CAA AT-3′), EF1α reverse (5′-GCC GTG TGG CAA TCC AAT-3′), MYB forward (5′-GCC AAT TAT CTC CCG AAT CGA-3′), MYB reverse (5′-ACC AAC GTT TCG GAC CGT A-3′), CSFR1 forward (5′-TCC AAC ATG CCG GCA ACT A-3′), CSFR1 reverse (5′-GCT CAA GTT CAA GTA GGC ACT CTC T-3′), CCR2 forward (5′-GAC AGA GAC TCT TGG GAT GAC TCA-3′), CCR2 reverse (5′-ATC CTA CAG CCA AGA GCT ATG TGA-3′), FLT3 forward (5′-CAA ATC AGA TGT ACG TGG AC-3′), FLT3 reverse (5′-GCT GTA TCC GTT ATC AAG AC-3′), MAF forward (5′-GTA CAA GGA GAA ATA CGA GAA G-3′), and MAF reverse (5′-TAT GAA AAA CTC GGG AGA GG-3′). Actin-β control forward and reverse primers were purchased from Eurogentech.

Immunostaining

Day 14 differentiated EBs were fixed in 4% paraformaldehyde, permeabilized in PBS + 0.3% Triton X-100 (Sigma), and blocked for 1 hr in blocking buffer (PBS + 10 μg/mL human serum IgG + 5% FBS). Samples were incubated overnight at 4°C in blocking buffer containing primary antibodies directly conjugated to FITC or APC. Antibodies used were α-CD43-FITC (1G10; BD Biosciences; 560978) and α-CD41a-APC (HIP8; BD Biosciences; 561852). Imaging was performed using an EVOS FL Auto Cell Imaging System using a ×10 or ×20 objective.

SUPPLEMENTAL INFORMATION

Supplemental Information includes Supplemental Experimental Procedures and five figures and can be found with this article online at http://dx.doi.org/10.1016/j.stemcr.2016.12.020.

AUTHOR CONTRIBUTIONS

J.B. designed, performed, and analyzed experiments and wrote the manuscript; W.J. and M.D.M. designed and supervised the studies and edited the manuscript.

ACKNOWLEDGMENTS

J.B. was funded by the RCUK–Medical Research Council and the Heatley Merck Sharpe and Dohme studentship. The Oxford Martin School (LC0910-004) and the Wellcome Trust (WTISSF121302) provide core support to the James Martin Stem Cell Facility within the Sir William Dunn School of Pathology. Samples and associated clinical data were supplied by the Oxford Parkinson's Disease Centre study, funded by the Monument Trust Discovery Award from Parkinson's UK, a charity registered in England and Wales (2581970) and in Scotland (SC037554), with the support of the National Institute for Health Research (NIHR) Oxford Biomedical Research Centre based at Oxford University Hospitals NHS Trust and University of Oxford, and the NIHR Comprehensive Local

Research Network. The High-Throughput Genomics Group at the Wellcome Trust Centre for Human Genetics (generation of Illumina genotyping) is funded by Wellcome Trust Grant Reference 090532/Z/09/Z.

Received: August 15, 2016
Revised: December 19, 2016
Accepted: December 20, 2016
Published: January 19, 2017

REFERENCES

Ackermann, M., Liebhaber, S., Klusmann, J.-H., and Lachmann, N. (2015). Lost in translation: pluripotent stem cell-derived hematopoiesis. EMBO Mol. Med. *7*, 1388–1402.

Anderson, K.L., Smith, K.A., Conners, K., Mckercher, S.R., Maki, R.A., Bruce, E., Anderson, B.K.L., and Torbett, B.E. (1998). Myeloid development is selectively disrupted in PU.1 null mice karen. Blood *91*, 3702–3710.

Aziz, A., Soucie, E., Sarrazin, S., and Sieweke, M.H. (2009). MafB/c-Maf deficiency enables self-renewal of differentiated functional macrophages. Science *326*, 867–871.

Bain, C.C., Bravo-Blas, A., Scott, C.L., Gomez Perdiguero, E., Geissmann, F., Henri, S., Malissen, B., Osborne, L.C., Artis, D., and Mowat, A.M. (2014). Constant replenishment from circulating monocytes maintains the macrophage pool in the intestine of adult mice. Nat. Immunol. *15*, 929–937.

Bigley, V., and Collin, M. (2011). Dendritic cell, monocyte, B and NK lymphoid deficiency defines the lost lineages of a new GATA-2 dependent myelodysplastic syndrome. Haematologica *96*, 1081–1083.

Bigley, V., Haniffa, M., Doulatov, S., Wang, X.-N., Dickinson, R., McGovern, N., Jardine, L., Pagan, S., Dimmick, I., Chua, I., et al. (2011). The human syndrome of dendritic cell, monocyte, B and NK lymphoid deficiency. J. Exp. Med. *208*, 227–234.

Chen, M.J., Yokomizo, T., Zeigler, B.M., Dzierzak, E., and Speck, N.A. (2009). Runx1 is required for the endothelial to haematopoietic cell transition but not thereafter. Nature *457*, 887–891.

Clarke, D., Vegiopoulos, A., Crawford, A., Mucenski, M., Bonifer, C., and Frampton, J. (2000). In vitro differentiation of c-myb(-/-) ES cells reveals that the colony forming capacity of unilineage macrophage precursors and myeloid progenitor commitment are c-Myb independent. Oncogene *19*, 3343–3351.

Cowley, S.A., Karlsson, K.R., Pelchen-Matthews, A., Miller, J., Moore, M.D., and James, W. (2012). Macrophages from human pluripotent stem cells. In Handbook of Macrophages: Life Cycle, Functions, and Diseases, R. Takahashi and H. Kai, eds. (Nova Science Publishers), pp. 83–120.

Epelman, S., Lavine, K.J., Beaudin, A.E., Sojka, D.K., Carrero, J.A., Calderon, B., Brija, T., Gautier, E.L., Ivanov, S., Satpathy, A.T., et al. (2014). Embryonic and adult-derived resident cardiac macrophages are maintained through distinct mechanisms at steady state and during inflammation. Immunity *40*, 91–104.

Flynn, R., Grundmann, A., Renz, P., Hänseler, W., James, W.S., Cowley, S.A., and Moore, M.D. (2015). CRISPR-mediated genotypic and phenotypic correction of a chronic granulomatous disease mutation in human iPS cells. Exp. Hematol. *43*, 838–848.

Gekas, C., Dieterlen-Lièvre, F., Orkin, S.H., Mikkola, H.K.A., and Marne, N. (2005). Hematopoietic stem cells. Am. J. Pathol. *8*, 365–375.

Ginhoux, F., Greter, M., Leboeuf, M., Nandi, S., See, P., Gokhan, S., Mehler, M.F., Conway, S.J., Ng, L.G., Stanley, E.R., et al. (2010). Fate mapping analysis reveals that adult microglia derive from primitive macrophages. Science *330*, 841–845.

Gomez Perdiguero, E., Klapproth, K., Schulz, C., Busch, K., Azzoni, E., Crozet, L., Garner, H., Trouillet, C., de Bruijn, M.F., Geissmann, F., et al. (2015). Tissue-resident macrophages originate from yolk-sac-derived erythro-myeloid progenitors. Nature *518*, 547–551.

Haniffa, M., Ginhoux, F., Wang, X., Bigley, V., Abel, M., Dimmick, I., Bullock, S., Grisotto, M., Booth, T., Taub, P., et al. (2009). Differential rates of replacement of human dermal dendritic cells and macrophages during hematopoietic stem cell transplantation. J. Exp. Med. *206*, 371–385.

Hashimoto, D., Chow, A., Noizat, C., Teo, P., Beasley, M.B., Leboeuf, M., Becker, C.D., See, P., Price, J., Lucas, D., et al. (2013). Tissue-resident macrophages self-maintain locally throughout adult life with minimal contribution from circulating monocytes. Immunity *38*, 792–804.

Hoeffel, G., Chen, J., Lavin, Y., Low, D., Almeida, F.F., See, P., Beaudin, A.E., Lum, J., Low, I., Forsberg, E.C., et al. (2015). C-Myb$^+$ erythro-myeloid progenitor-derived fetal monocytes give rise to adult tissue-resident macrophages. Immunity *42*, 665–678.

Ivanovs, A., Rybtsov, S., Welch, L., Anderson, R.A., Turner, M.L., and Medvinsky, A. (2011). Highly potent human hematopoietic stem cells first emerge in the intraembryonic aorta-gonad-mesonephros region. J. Exp. Med. *208*, 2417–2427.

Jiang, Y., Cowley, S.A., Siler, U., Melguizo, D., Tilgner, K., Browne, C., Dewilton, A., Przyborski, S., Saretzki, G., James, W.S., et al. (2012). Derivation and functional analysis of patient-specific induced pluripotent stem cells as an in vitro model of chronic granulomatous disease. Stem Cells *30*, 599–611.

Kanitakis, J., Morelon, E., Petruzzo, P., Badet, L., and Dubernard, J.M. (2011). Self-renewal capacity of human epidermal Langerhans cells: observations made on a composite tissue allograft. Exp. Dermatol. *20*, 145–146.

Karlsson, K.R., Cowley, S., Martinez, F.O., Shaw, M., Minger, S.L., and James, W. (2008). Homogeneous monocytes and macrophages from human embryonic stem cells following coculture-free differentiation in M-CSF and IL-3. Exp. Hematol. *36*, 1167–1175.

Kierdorf, K., Erny, D., Goldmann, T., Sander, V., Schulz, C., Perdiguero, E.G., Wieghofer, P., Heinrich, A., Riemke, P., Hölscher, C., et al. (2013). Microglia emerge from erythromyeloid precursors via Pu.1- and Irf8-dependent pathways. Nat. Neurosci. *16*, 273–280.

Klimchenko, O., Di Stefano, A., Geoerger, B., Hamidi, S., Opolon, P., Robert, T., Routhier, M., El-Benna, J., Delezoide, A.-L., Boukour, S., et al. (2011). Monocytic cells derived from human embryonic stem cells and fetal liver share common differentiation pathways and homeostatic functions. Blood *117*, 3065–3075.

Lahmar, Q., Keirsse, J., Laoui, D., Movahedi, K., Van Overmeire, E., and Van Ginderachter, J.A. (2015). Tissue-resident versus monocyte-derived macrophages in the tumor microenvironment. Biochim. Biophys. Acta Rev. Cancer *1865*, 23–34.

Lieu, Y.K., and Reddy, E.P. (2009). Conditional c-myb knockout in adult hematopoietic stem cells leads to loss of self-renewal due to impaired proliferation and accelerated differentiation. Proc. Natl. Acad. Sci. USA *106*, 21689–21694.

Litvack, M.L., Wigle, T.J., Lee, J., Wang, J., Ackerley, C., Grunebaum, E., and Post, M. (2016). Alveolar-like stem cell-derived myb(neg) macrophages promote recovery and survival in airway disease. Am. J. Respir. Crit. Care Med. *193*, 1–69.

Mucenski, M.L., McLain, K., Kier, A.B., Swerdlow, S.H., Schreiner, C.M., Miller, T.A., Pietryga, D.W., Scott, W.J., and Potter, S.S. (1991). A functional c-myb gene is required for normal murine fetal hepatic hematopoiesis. Cell *65*, 677–689.

Mukouyama, Y.S., Chiba, N., Mucenski, M.L., Satake, M., Miyajima, A., Hara, T., and Watanabe, T. (1999). Hematopoietic cells in cultures of the murine embryonic aorta-gonad-mesonephros region are induced by c-Myb. Curr. Biol. *9*, 833–836.

Okada, H., Watanabe, T., Niki, M., Takano, H., Chiba, N., Yanai, N., Tani, K., Hibino, H., Asano, S., Mucenski, M.L., et al. (1998). AML1(-/-) embryos do not express certain hematopoiesis-related gene transcripts including those of the PU.1 gene. Oncogene *17*, 2287–2293.

Okuda, T., Van Deursen, J., Hiebert, S.W., Grosveld, G., and Downing, J.R. (1996). AML1, the target of multiple chromosomal translocations in human leukemia, is essential for normal fetal liver hematopoiesis. Cell *84*, 321–330.

Palis, J., Robertson, S., Kennedy, M., Wall, C., and Keller, G. (1999). Development of erythroid and myeloid progenitors in the yolk sac and embryo proper of the mouse. Development *126*, 5073–5084.

Palis, J., Chan, R.J., Koniski, A., Patel, R., Starr, M., and Yoder, M.C. (2001). Spatial and temporal emergence of high proliferative potential hematopoietic precursors during murine embryogenesis. Proc. Natl. Acad. Sci. USA *98*, 4528–4533.

Rafii, S., Kloss, C.C., Butler, J.M., Ginsberg, M., Gars, E., Lis, R., Zhan, Q., Josipovic, P., Ding, B.-S., Xiang, J., et al. (2013). Human ESC-derived hemogenic endothelial cells undergo distinct waves of endothelial to hematopoietic transition. Blood *121*, 770–780.

Rückerl, D., and Allen, J.E. (2014). Macrophage proliferation, provenance, and plasticity in macroparasite infection. Immunol. Rev. *262*, 113–133.

Sandor, C.R.P., Lang, C., Heger, A., Booth, H., Vowles, J., Witty, L., Bowden, R., Hu, M., Cowley, S.A., Wade-Martins, R., and Webber, C. (2017). Transcriptomic profiling of purified patient-derived dopamine neurons identifies convergent perturbations and therapeutics for Parkinson's disease. Hum. Mol. Genet. http://dx.doi.org/10.1093/hmg/ddw412.

Schulz, C., Gomez Perdiguero, E., Chorro, L., Szabo-Rogers, H., Cagnard, N., Kierdorf, K., Prinz, M., Wu, B., Jacobsen, S.E.W., Pollard, J.W., et al. (2012). A lineage of myeloid cells independent of Myb and hematopoietic stem cells. Science *336*, 86–90.

Scott, E.W., Simon, M.C., Anastasi, J., and Singh, H. (1994). Requirement of transcription factor PU.1 in the development of multiple hematopoietic lineages. Science *265*, 1573–1577.

Sheng, J., Ruedl, C., and Karjalainen, K. (2015). Most tissue-resident macrophages except microglia are derived from fetal hematopoietic stem cells. Immunity *43*, 382–393.

Sumner, R., Crawford, A., Mucenski, M., and Frampton, J. (2000). Initiation of adult myelopoiesis can occur in the absence of c-Myb whereas subsequent development is strictly dependent on the transcription factor. Oncogene *19*, 3335–3342.

Tamoutounour, S., Guilliams, M., MontananaSanchis, F., Liu, H., Terhorst, D., Malosse, C., Pollet, E., Ardouin, L., Luche, H., Sanchez, C., et al. (2013). Origins and functional specialization of macrophages and of conventional and monocyte-derived dendritic cells in mouse skin. Immunity *39*, 925–938.

van de Laar, L., Saelens, W., De Prijck, S., Martens, L., Scott, C.L., Van Isterdael, G., Hoffmann, E., Beyaert, R., Saeys, Y., Lambrecht, B.N., and Guilliams, M. (2016). Yolk sac macrophages, fetal liver, and adult monocytes can colonize an empty niche and develop into functional tissue-resident macrophages. Immunity *44*, 755–768.

Vanhee, S., De Mulder, K., Van Caeneghem, Y., Verstichel, G., Van Roy, N., Menten, B., Velghe, I., Philippe, J., De Bleser, D., Lambrecht, B.N., et al. (2015). In vitro human embryonic stem cell hematopoiesis mimics MYB-independent yolk sac hematopoiesis. Haematologica *100*, 157–166.

van Wilgenburg, B., Browne, C., Vowles, J., and Cowley, S.A. (2013). Efficient, long term production of monocyte-derived macrophages from human pluripotent stem cells under partly-defined and fully-defined conditions. PLoS One *8*, e71098.

van Wilgenburg, B., Moore, M.D., James, W.S., and Cowley, S.A. (2014). The productive entry pathway of HIV-1 in macrophages is dependent on endocytosis through lipid rafts containing CD4. PLoS One *9*, 1–14.

van Wilgenburg, B., Scherwitzl, I., Hutchinson, E.C., Leng, T., Kurioka, A., Kulicke, C., de Lara, C., Cole, S., Vasanawathana, S., Limpitikul, W., et al. (2016). MAIT cells are activated during human viral infections. Nat. Commun. *7*, 11653.

Wang, Q., Stacy, T., Binder, M., Marin-Padilla, M., Sharpe, A.H., and Speck, N.A. (1996). Disruption of the Cbfa2 gene causes necrosis and hemorrhaging in the central nervous system and blocks definitive hematopoiesis. Proc. Natl. Acad. Sci. USA *93*, 3444–3449.

Zakrzewska, A., Cui, C., Stockhammer, O.W., Benard, E.L., Spaink, H.P., and Meijer, A.H. (2010). Macrophage-specific gene functions in Spi1-directed innate immunity. Blood *116*, e1–11.

Stem Cell Reports, Volume *8*

Supplemental Information

Human Induced Pluripotent Stem Cell-Derived Macrophages Share Ontogeny with *MYB*-Independent Tissue-Resident Macrophages

Julian Buchrieser, William James, and Michael D. Moore

Supplementary Figures and legends

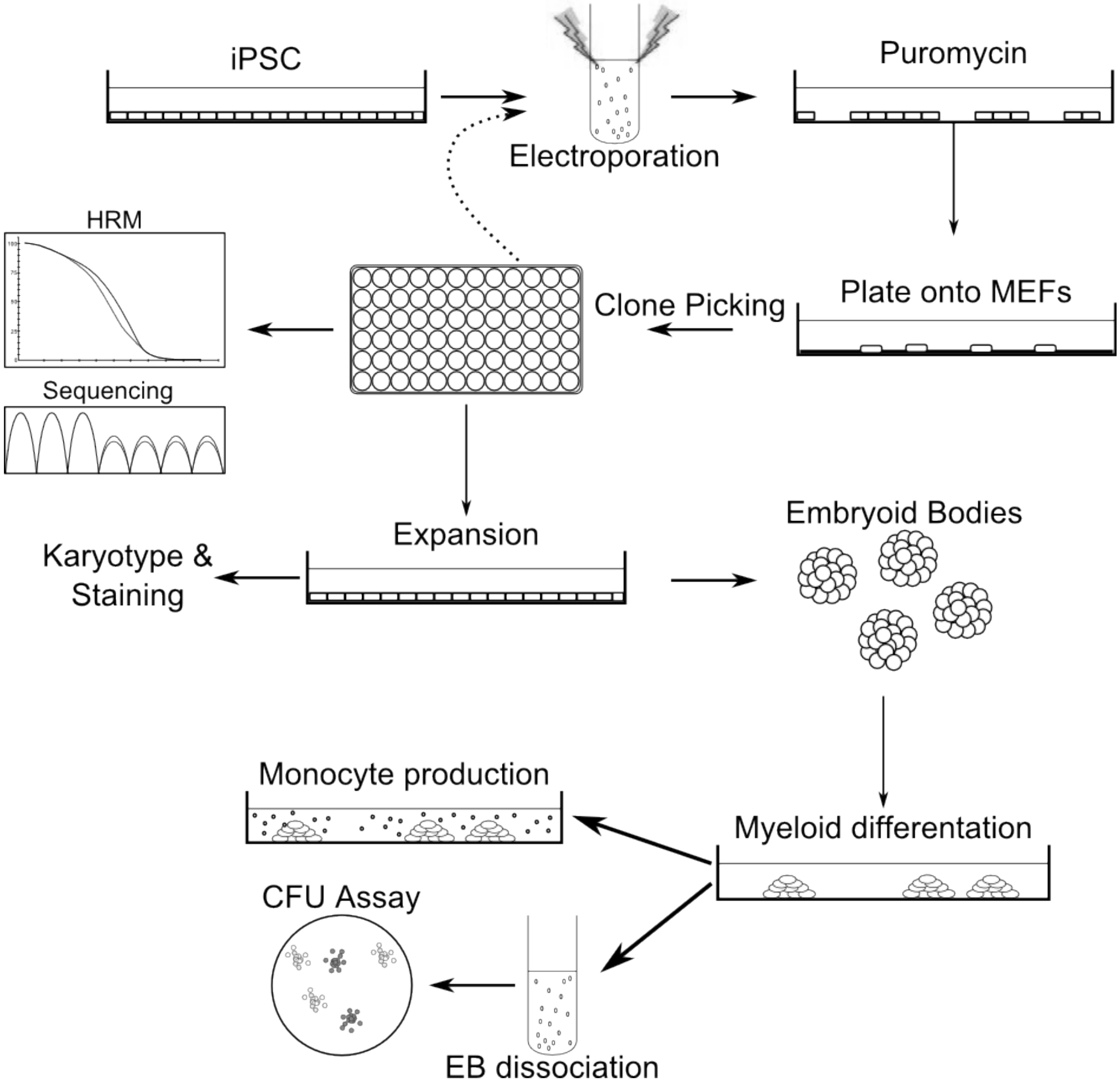

Supplementary figure S1. Schematic representation of the CRISPR-Cas9 based knock-out strategy in iPSCs. *Related to supplementary methods "Gene editing and single-cell cloning".* IPSCs generated from a healthy donor were cultured under feeder-free conditions, electroporated with two, pX462 plasmids for Cas9-gRNA-puromycin expression. Transfected cells were selected by transient puromycin treatment after which they were plated at low density onto mouse embryonic fibroblast feeder cells. After 7 days of expansion, individual single cell colonies were picked into a 96 well plate directly in feeder-free conditions and clones were expanded. IPSC clones were pre-screened for insertions or deletions in the targeted region using high resolution melt analysis (HRM)

(Supplementary Figure S2A) and sequenced to determine the exact sequence of each clone (Supplementary Figure S3C). Clones harbouring a single out of frame or a double out of frame deletion in the gene of interest were expanded, stained for pluripotency markers and karyotyped (Supplementary Figure S4). IPSC lines were then assessed for myeloid differentiation potential and hematopoietic colony formation capacity.

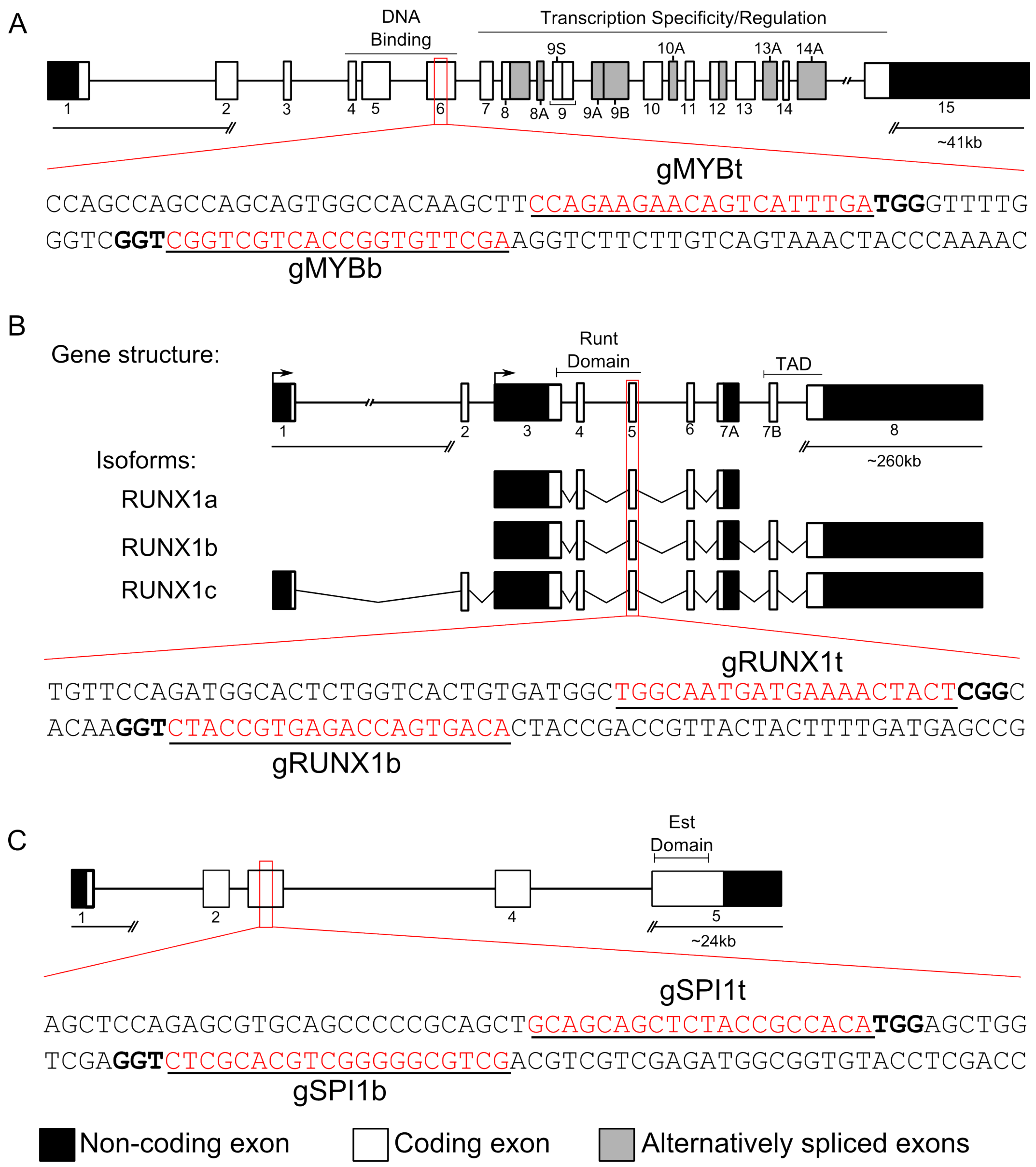

Supplementary figure S2. Exon structure, splice variants, major functional domains and CRISPR-Cas9 target site of the *MYB* (A), *RUNX1* (B) and *SPI1* (C) genes. *Related to supplementary methods "Gene editing and single-cell cloning".* For each gene an enhanced view of the target sequence is shown, CRISPR-Cas9 targeting site is shown in red and PAM sites in bold. *RUNX1* encodes for three major isoforms, RUNX1a, RUNX1b and RUNX1c (Osato, 2014).

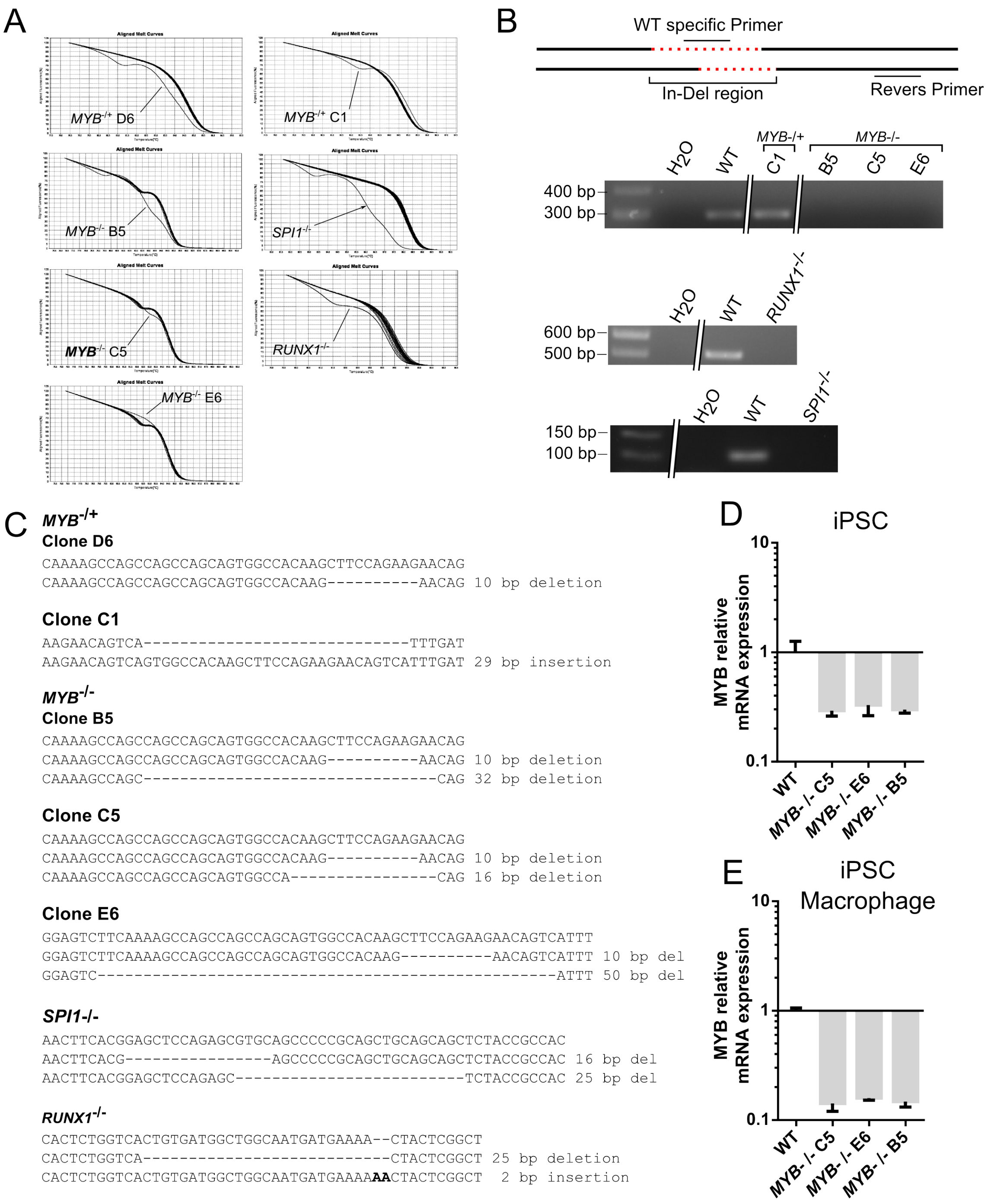

Supplementary figure S3. Genomic analysis of the knock-out iPSC clones. *Related to supplementary methods "Gene editing and single-cell cloning".* (**A**) High resolution melt curves obtained from the 7 different iPSC clones used in this study shown in comparison to the melt curves obtained from their respective unmodified parent lines. (**B**) Primers designed to specifically amplify wild type sequence and not knock-out sequence showed

total absence of amplification in all knock-out clones. First, a schematic representation of WT specific primer design, forward primers were designed to span the deletion and/or insertion present in the knock-out clones hindering binding of the primers and amplification of knock-out sequence but allowing the amplifying WT sequence. Second, *MYB* clones were tested using forward primer JB-107 (5'-TGGCCACAAGCTTCCAGAAG-3') and reverse primer JB-64 (5'-ACCATACCTACACCCTATCTACTTCAAAG-3'). *MYB* and WT clones amplified while all three *MYB* clones did not amplify any product. Third, *RUNX1* clone was tested using forward primer JB-110 (5'-GGCTGGCAATGATGAAACCT-3') and reverse primer JB-78 (5'-GATAGCCCACAGATCATACGTCAA-3'), the forward primer was designed with an extra mismatch to increase specificity as one allele of the *RUNX1* clone is a short 2 bp insertion. WT positive control DNA amplified while *RUNX1* didn't. Last, *SPI1* clone was tested, forward primer JB-108 (5'-GAGCTCCAGAGCGTGCAGCC-3') and reverse primer JB-92 (5'-CAGGAGGGCCCACAACAA-3'). WT positive control DNA amplified while *SPI1* didn't. (**C**) Sequence analysis of the different knock-out single iPSC clones showing both alleles compared to the original wild type sequence. Each complete knock-out clone harbours an out-of-frame deletion or insertion on both alleles, while single allele knock-out clone still harbour one wild type allele. (**D-E**) Relative expression of *MYB* mRNA in WT and *MYB*$^{-/-}$ iPSCs and iPSC-derived monocytes/macrophages showing an efficient knock-down of the mRNA levels of *MYB*.

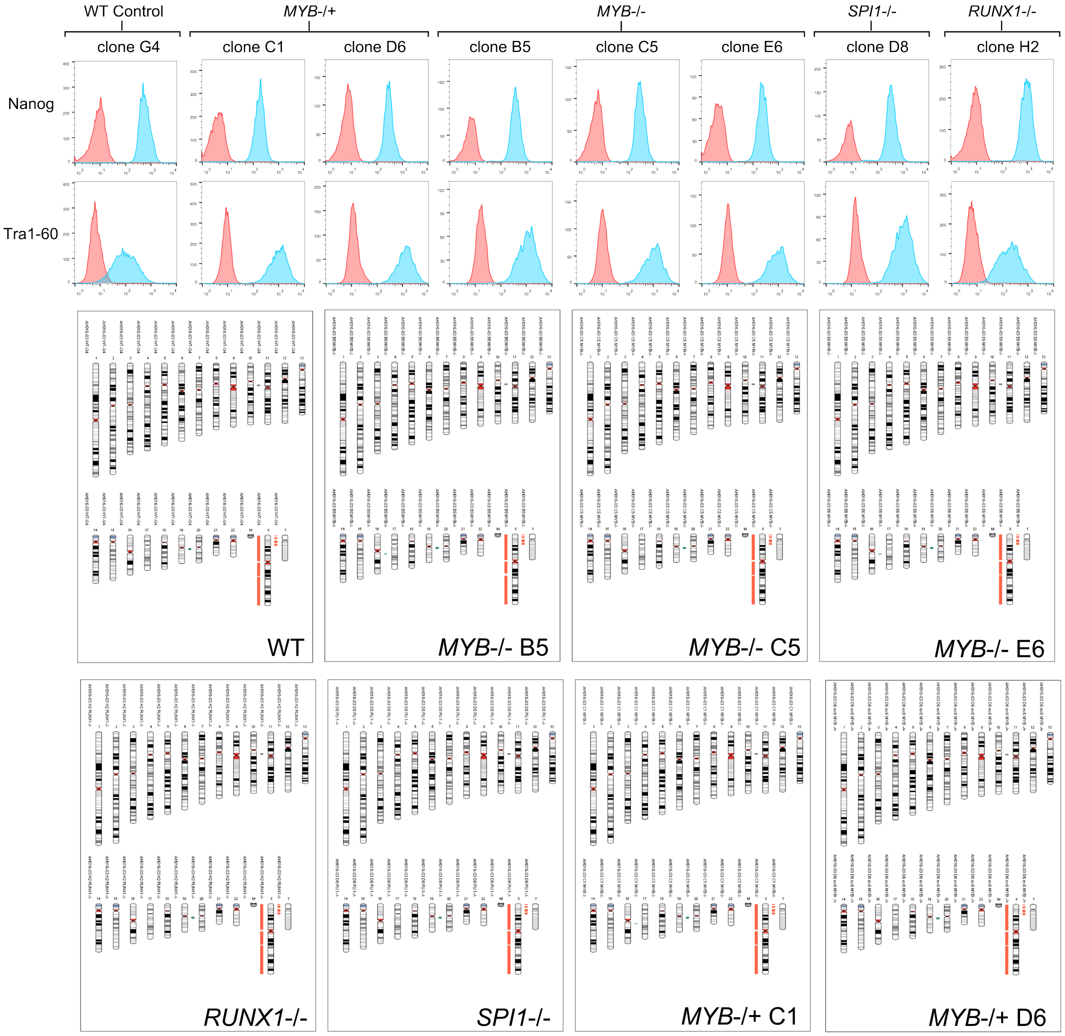

Supplementary figure S4. Gene-knock-out iPSC lines maintained pluripotency and karyotype. *Related to supplementary methods "Gene editing and single-cell cloning".* (**A**) iPSCs lines were stained for TRA-1-60 (1.5 mg/mL; α-TRA-1-60-AlexaFluor®488; Biolegend; 330614) and NANOG (0.3 mg/mL; α-NANOG-AlexaFluor®647; Cell Signaling Technologies; D73G4), flow cytometry analysis are shown as histograms, antibody staining (blue) and isotype (red). (**B**) DNA extracted from the iPSCs was karyotyped using a SNP array (Illumina OmniExpress24 chip covering w700,000 markers) and analysed using KaryoStudio (Illumina) to detect copy number variations across the genome. Red indicates a single copy of the SNPs (demonstrated by the single X copy in this male patient's DNA); gray indicates loss of heterozygosity; and green indicates duplications of a stretch of DNA (one amplification on ch20 was present on all iPSC clones).

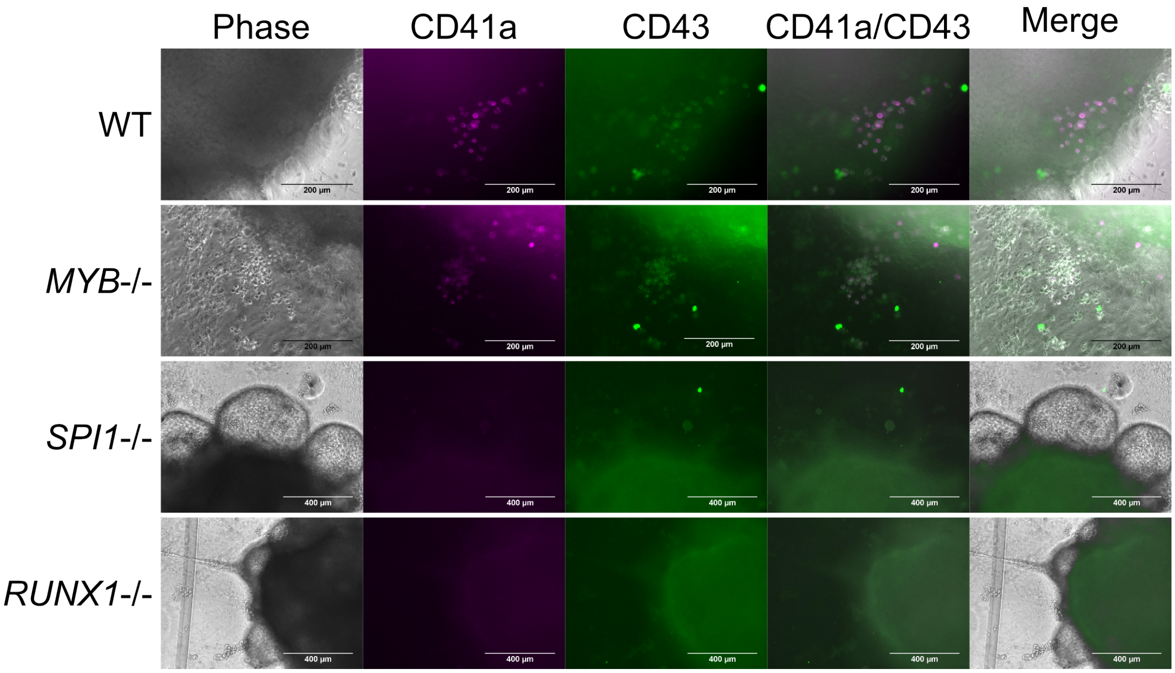

Supplementary figure S5. CD41a$^+$CD43$^+$ early progenitors are detected in WT and MYB-/- EBs. *Related to Figure 4.* WT, *MYB*$^{-/-}$, *RUNX1*$^{-/-}$ and *SPI1*$^{-/-}$ day 14 EBs were stained for CD41a and CD43 hematopoietic markers and imaged on a EVOS FL Auto Cell Imaging System. CD41a$^+$CD43$^+$ early hematopoietic progenitors can be detected in both *MYB*$^{-/-}$ and WT iPSC differentation while they are completley absent in *SPI1*$^{-/-}$ and *RUNX1*$^{-/-}$ iPSC differentation.

Supplemental experimental procedures

Vector construction

The CRISPR-Cas9 vectors used in this study were based on the dual Cas9- and guide RNA (gRNA)-, puromycin-resistance gene-expressing, pSpCas9n(BB)-2A-Puro (pX462) vector (Cong et al., 2013) (gift from Feng Zhang (Addgene plasmid #48141). Cloning was performed as previously described (Cong et al., 2013) using oligonucleotides JB-73 (5' CACCGGCTTGTGGCCACTGCTGGC 3') and JB-74 (5' AAACGCCAGCA-GTGGCCACAAGCC 3') with pX462 to create pX462-gMYBt; oligonucleotides JB-75 (5' CACCGCAGAAGAACAGTCATTTGA 3') and JB-76 (5' AAACTCAAAT-

GACTGTTCTTCTGC 3’) with pX462 to create pX462-gMYBb; oligonucleotides JB-81 (5’ CACCGCAGTGACCAGAGTGCCATC 3’) and JB-82 (5’ AAACGATGGCACTCT-GGTCACTGC 3’) with pX462 to create pX462-gRUNX1t; oligonucleotides JB-83 (5’ CACCGGGCAATGATGAAAACTACT 3’) and JB-84 (5’ AAACAGTAGT-TTTCATCATTGCCC 3’) with pX462 to create pX462-gRUNX1b; oligonucleotides JB-101 (5’ CACCGCTGCGGGGGCTGCACGCTC 3’) and JB-102 (5’ AAACGAGC-GTGCAGCCCCCGCAGC 3’) with pX462 to create pX462-gSPI1t; and oligonucleotides JB-103 (5’ CACCGCAGCAGCTCTACCGCCACA 3’) and JB-104 (5’ AAACTG-TGGCGGTAGAGCTGCTGC 3’) with pX462 to create pX462-gSPI1b.

Gene editing and single-cell cloning

Knock-out iPSC lines of *MYB*, *RUNX1* and *SPI1* were generated using a dual guide RNA (gRNA)-targeting strategy (Supplementary Figure S1). The location and sequence of the gRNA pairs designed for targeting the *MYB*, *RUNX1* and *SPI1* genes are shown in Supplementary Figure S2. $2x10^6$ feeder-free iPSCs were transfected with two pX462 plasmids in a single-cell suspension by electroporation (Neon®transfection system, Invitrogen), using a 100μL tip with 10μg DNA (5μg top strand pX462-gRNA and 5 μg bottom strand pX462-gRNA). After one pulse of electroporation at 1000 volts and 40 ms pulse width, $1x10^6$ transfected cells were plated onto a matrigel coated 12 well plate in mTeSR1 supplemented with 10 μmol/L Y-27632 without penicillin/streptomycin. After 48h, cells were puromycin selected (0.4 μg/mL; MP Biomedicals) for 48h. Surviving cells were plated at 10^4 cells per 10 cm dish on mitotically-inactivated mouse embryonic fibroblast feeder cells (MEF; outbred Swiss mice established and maintained at the Department of Pathology, Oxford (Chia et al., 2005; Gardner, 1982)) on gelatin-coated tissue culture plates in hESC medium (KO-DMEM, 2 mmol/L L-Glutamine, 100 mmol/L non-essential amino acids, 20% serum replacement, and 8 ng/mL basic fibroblastic growth factor (FGF2)), supplemented with 10 μmol/L Y-27632 on the day of the plating. After 7 days of expansion, individual single-cell colonies were picked manually onto a matrigel coated 96 well plate in mTeSR1. Clones were expanded and pre-screened using high-resolution melt analysis (HRM) on a StepOnePlus Real-Time PCR System

(ThermoFisher). AmpliTaq®Gold DNA Polymerase (ThermoFisher) was used with LCGreen Plus+ (BioChem) melting dye. The following primers were used: JB-71 (5' ACAGGAAGGTTATCTGCAGGAGTCT 3') + JB-72 (5' AGTGGCAGGG-AGTTGAGCTGTA 3') for *MYB*, JB-79 (5' ATCACTACACAAATGCCCTAAAAGTG 3') + JB-80 (5' TTAAATCTTGCAACCTGGTTCTTCA 3') for *RUNX1* and JB-99 (5' CAGACCATTACTGGGACTTCCA 3') + JB-100 (5' GGGTATCGAGGACGTGCATCT 3') for *SPI1*. Genetically modified clones detected by HRM (Supplementary Figure S3A) were sequenced and analysed for insertions and/or deletions, after which, double and single knock-out iPSC clones were expanded and characterized. The first round of transfection produced several single out-of-frame deletion *MYB* clones but did not result in any double out-of-frame knock-out clones. We therefore used a single out-of-frame knock-out clone, clone D6, for re-targeting. Clone D6 was expanded and transfected with the two pX462 Cas9-gRNA-puromycin expressing plasmids targeting *MYB* and processed as previously. After a second round of targeting, several out-of frame knock-outs were generated and 3 clones (Clone B5, C5 and E6) were used in this study. Sequence analysis of the three *MYB*$^{-/-}$, two *MYB*$^{+/-}$, single *RUNX1*$^{-/-}$ and single *SPI1*$^{-/-}$ iPSC clones are shown in Supplementary Figure S3C. After clonal expansion, presence of wild-type cells in the single-cell clone knock-out iPSC lines was excluded by PCR (Supplementary Figure S3B) The cell clones showed normal undifferentiated morphology, expressed pluripotency markers Tra-1-60 and Nanog and no gross karyotypic abnormalities were detected by SNP array (Supplementary Figure S4).

Supplementary references

Chia, R., Achilli, F., Festing, M.F., Fisher, E.M., 2005. The origins and uses of mouse outbred stocks. Nat Genet 37, 1181–1186.

Cong, L., Ran, F.A., Cox, D., Lin, S., Barretto, R., Habib, N., Hsu, P.D., Wu, X., Jiang, W., Marraffini, L.A., Zhang, F., 2013. Multiplex Genome Engineering Using CRISPR/Cas System. Science. 339, 403–406.

Gardner, R.L., 1982. Investigation of cell lineage and differentiation in the extraembryonic endoderm of the mouse embryo. J. Embryol. Exp. Morphol. 68, 175–198.

Osato, M., 2014. An unsung runt 6e isoform for HSC expansion. Blood 123, 3684–3686.
